# Synergistic metalloproteinase-based remodeling of matrix by pancreatic tumor and stromal cells

**DOI:** 10.1371/journal.pone.0248111

**Published:** 2021-03-19

**Authors:** Hong Cao, Li Qiang, Jing Chen, Katherine M. Johnson, Mark A. McNiven, Gina L. Razidlo

**Affiliations:** 1 Division of Gastroenterology & Hepatology, Mayo Clinic, Rochester, Minnesota, United States of America; 2 Department of Biochemistry & Molecular Biology, Mayo Clinic, Rochester, Minnesota, United States of America; University of Liverpool, UNITED KINGDOM

## Abstract

The process by which tumor cells mechanically invade through the surrounding stroma into peripheral tissues is an essential component of metastatic dissemination. Matrix metalloproteinase (MMP)-mediated extracellular matrix (ECM) degradation plays an important role in this invasive process. Defining the contribution and interaction between these MMPs during invasion remains a key interest in the development of targeted anti-metastatic therapies. In this study we have utilized multiple different stromal fibroblasts and tumor cells to define the relative contributions between cancer cells and stromal cells during MMP-dependent matrix remodeling and pancreatic (PDAC) tumor cell invasion. We find that tumor cells co-cultured with the conditioned medium from stromal fibroblasts exhibited a substantial increase in invadopodial-based matrix degradation and transwell invasion. This increase is dependent on pro-MMP2 expressed and secreted by stromal fibroblasts. Further, the pro-MMP2 from the stromal fibroblasts is activated by MT1-MMP expressed on the tumor cells. Depletion of MT1-MMP, the known activator of MMP2, in tumor cells largely blocked matrix remodeling, even in the presence of stromal cell medium. In summary, these findings implicate an important interplay between MT1-MMP from tumor cells and MMP2 from fibroblasts as a key component for ECM remodeling and invasion.

## Introduction

Pancreatic ductal adenocarcinoma (PDAC) is a lethal and highly metastatic cancer. The high mortality rate for pancreatic cancer is largely due to the advanced stage at which the disease is diagnosed, resistance to therapies, and particularly early metastasis [1–3]. Pancreatic tumors are exceptionally invasive and are believed to invade peripheral tissues via a combination of stromal remodeling and active migration that facilitates the intravasation of this tumor into the adjacent vasculature [4, 5]. This remodeling of the surrounding tumor environment is achieved in part through the secretion of a variety of different matrix metalloproteinases (MMPs).

Various types of invasive and metastatic cancer exhibit high levels of MMPs, such as prostate, breast, ovarian, colorectal and pancreatic cancers [6, 7]. Currently, a total of 24 MMPs have been identified, comprising 18 secreted matrix metalloproteinases and 6 membrane-type matrix metalloproteinases [8]. Several of these MMPs are upregulated in pancreatic cancer, particularly MT1-MMP, MMP2 and MMP9 [9–11]. MMPs are well known to act at subcellular sites termed invadopodia [5, 12–14], which are cytoskeletal protrusions at the cell surface that extend into the surrounding tumor matrix. These cytoskeletal structures are complex membrane specializations comprised of many cytoskeletal scaffold proteins and regulatory enzymes situated along an actin-rich core [13]. One specific protease, MT1-MMP, is well documented to reside at invadopodia where it acts to degrade the extracellular matrix from these structures.

Recently, the stromal cells in the tumor microenvironment have been shown to possess a matrix remodeling capacity that can work synergistically with adjacent tumor cells to facilitate invasive dissemination [15–17]. Indeed, it well documented that activated stromal fibroblasts drive the deposition of significant extracellular matrix making PDAC markedly desmoplastic [18, 19]. These matrix secreting cancer-associated fibroblasts (CAFs) are heterogeneous and may represent distinct populations that are pro-fibrotic and/or pro-inflammatory [20, 21]. Pancreatic stellate cells (PSCs) represent a source of CAFs in the pancreatic tumor microenvironment that can provide paracrine signals, metabolites, and lipids that crosstalk with the epithelial tumor cells [20]. Stromal fibroblasts contribute to tumor cell invasion directly as they have a capacity to degrade and remodel the extracellular matrix via the secretion of MMPs [5, 22, 23]. These CAFs are more contractile than normal fibroblasts, and can also assemble and align the fibronectin rich extracellular matrix to promote cancer cell migration [24–26]. CAFs also facilitate tumor cell invasion through mechanical pressure and via the secretion of factors that act on the tumor cells to amplify tumor invasion including growth factors, MMPs, cytokines, and chemokines [21, 26–28]. Multiple types of MMPs are secreted by CAFs to facilitate ECM remodeling, such as MMP1, MMP2, MMP9, and MMP13 [29–31]. MMP2 in particular is secreted by stromal cells, including PSCs [32, 33]. In addition to remodeling the extracellular matrix, secreted MMPs can also target and activate tumor cell signaling pathways. For example, MMP1 secreted by fibroblasts can promote breast cancer cell migration and invasion by cleaving protease-activated receptor 1 (PAR1) and generating PAR1-dependent Ca^2+^ signals [34]. In pancreatic cancer, the activation of MMP2 by MT1-MMP has been directly related to its progression and invasion [35]. However, information on the origin, biology, and contribution of these stromal-derived trans-acting factors with regard to pancreatic cancer invasion, and their mechanisms of cross-talk with tumor cells, remains to be further defined.

The goal of this study was to investigate the functional interaction between PDAC tumor cells and associated stromal fibroblasts with regard to remodeling of the surrounding extracellular matrix. We have specifically focused on pancreatic stellate cells and normal fibroblasts to investigate crosstalk in the earliest stages of tumorigenesis, where pancreatic tumor cell dissemination has been reported to occur [2]. We have observed that PDAC cells incubated with conditioned medium from stromal fibroblasts form an increased number of functional invadopodia, resulting in an increased capacity to degrade a gelatin substrate as well as invade *in vitro*. This increased invasiveness is dependent upon a synergistic interaction between MT1-MMP residing within the tumor cells that acts to degrade the surrounding matrix while also activating MMP2, which is secreted from nearby fibroblasts in an inactive form. These findings provide new insights into the functional cooperation between PDAC cells and the surrounding CAFs toward stromal remodeling during metastatic invasion.

## Results

### Fibroblasts stimulate the invasive properties of pancreatic cancer cells

**Fig 1:**
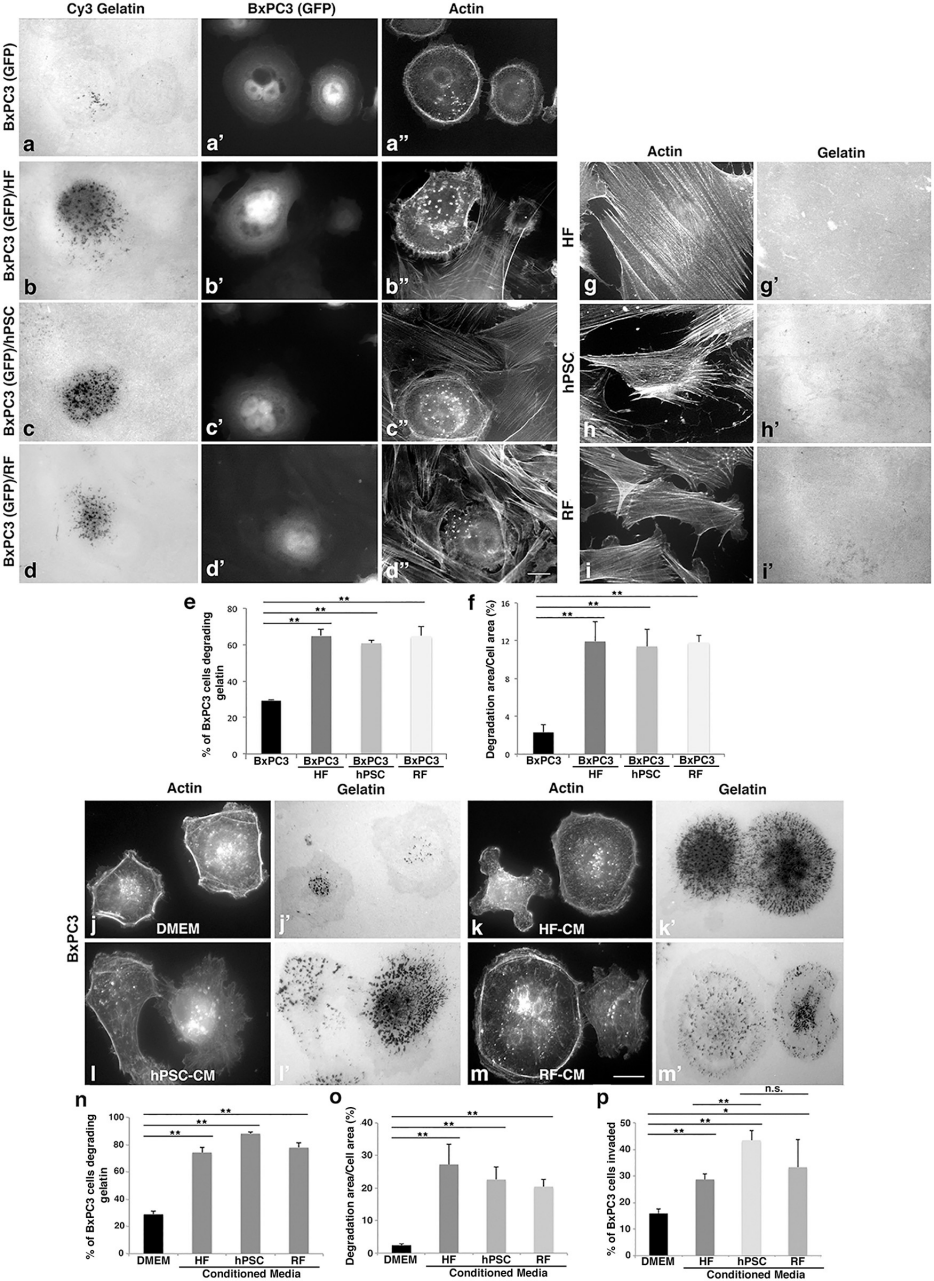
Fibroblasts stimulate the invasive properties of pancreatic cancer cells. (a-f) ECM degradation by BxPC3 cells is increased by coculturing with fibroblasts. BxPC3 cells stably expressing GFP were seeded onto Cy3-fluorescent gelatin-coated coverslips alone (a, a’,a”) or together with HFs (b, b’, b”), hPSCs (c, c’, c”), or RFs (d, d’, d”). Cells were stained for actin and gelatin degradation was quantified after 8 h. Tumor cells were distinguished by GFP expression. Scale bar = 10μm. Co-culture with any of these stromal cells results in an over 2-fold increase in the percentage of cells degrading the gelatin substrate by BxPC3 cells (≥100 cells per condition, e) and a 6 fold increase in the area degraded per cell in BxPC3 cells (≥10 cells per condition, f). (g-i) Fibroblasts were plated on Cy3-fluorescent gelatin-coated coverslips and stained for actin using phalloidin. The HF (g, g’), hPSC (h, h’), and RF (i,i’), degrade minimal matrix after 8 hours. (j-o) Conditioned medium (CM) from fibroblasts promotes ECM degradation by PDAC cells. BxPC3 cells were seeded onto green fluorescent gelatin-coated coverslips with DMEM only (j, j’) or CM collected from HFs (k, k’), hPSCs (l, l’), and RF cells (m, m’). Cells were stained for actin and gelatin degradation was quantified after 8 h. Coculture of BxPC3 cells with CM from fibroblasts increased the percent of BxPC3 cells degrading the gelatin substrate by 2 fold (≥100 cells per condition, n) and the area degraded per cell in BxPC3 cells by 4–6 fold (≥10 cells per condition, o). (p) The conditioned medium from fibroblasts increased BxPC3 cell transwell invasion. BxPC3 cells were seeded in transwell invasion assays for 6 h with DMEM or CM from fibroblasts. The presence of CM from HF, hPSC, and RF all increased the number of BxPC3 cells migrating through the filters. All graphed data represent the mean ± SEM of 3 independent experiments. *p <0.05. **p <0.01. Scale bars = 10μm.

As fibroblasts are known to promote invasive metastasis of various tumor types by a variety of processes, we tested if stromal cells might alter the ability of pancreatic cancer cells to degrade extracellular matrix components *in vitro*. To this end, BxPC3 PDAC cells were seeded on fluorescent gelatin-coated coverslips alone, with or without human pancreatic stellate cells (hPSC), which are one source of CAFs in pancreatic tumors, as well as normal human or rat fibroblasts. To distinguish the tumor cells from the fibroblasts, the BxPC3 tumor cells were stably transfected with GFP (pEGFP N1) vector. The capacity of PDAC cells to degrade the surrounding substrate was quantified by both the percentage of PDAC cells degrading the matrix and the area of degradation per PDAC cell area. Importantly, coculture with stromal cells enhanced the percentage of BxPC3 cells degrading the matrix over 2 fold and the area of degradation per BxPC3 cell area 6–9 fold (Fig 1a–1f). The experiment was also performed using PANC-1 cells, which are normally unable to degrade surrounding substrates. Strikingly, the PANC-1 cells exhibited a substantial increase in both the percentage of cells capable of degrading gelatin and the area of degradation per PANC-1 cell area upon coculturing with fibroblasts (S1a–S1d Fig). Consistent with our prior studies, the hPSCs, HFs, and RFs did not degrade significant amounts of matrix at this timepoint when plated alone (Fig 1g–1i) [36]. These findings suggest that fibroblasts stimulate the invasive properties of pancreatic cancer cells.

To test if stromal cells contribute to tumor cell ECM degradation by a direct physical interaction or by the secretion of trans-acting factors, we collected serum-free conditioned medium (CM) from the hPSCs, HFs, or RFs. This media was applied to 4 different PDAC cell lines (BxPC3, PANC-1, DanG, HPAF-II cells) and the level of matrix degradation exhibited by these cells was quantitated. Incubation with stromal cell CM induced substantial levels of ECM degradation that mimicked the levels observed from a direct coculturing of the PDAC cells with fibroblasts directly (Fig 1j–1o, S1e–S1j and S2 Figs). These findings indicate that fibroblasts stimulate pancreatic cancer cell-mediated ECM degradation by secreting factors into the environment. The ECM remodeling capacity of MDA-MB-231 breast cancer cells is also promoted by CM from fibroblasts, suggesting that this important process is utilized by multiple tumor types (S2 Fig).

To extend the matrix degradation assays into a more biological context, the effects of CM collected from stromal cells were tested using transwell invasion assays. In these experiments BxPC3 cells were seeded in chemotactic transwell invasion chambers on the top side of porous filters coated with 0.3% gelatin and incubated in either serum-free DMEM control media or CM. Cell invasion from the top to the bottom chamber was initiated by the inclusion of 10% FBS in the media in the bottom chamber acting as a chemo-attractant. BxPC3 cells exhibited a 2–3 fold increase in invasion when incubated with fibroblast CM compared to the cells cultured with control DMEM only, particularly with the conditioned medium from the hPSCs (Fig 1p). Taken together these findings suggest that cancer cells can sense and respond to trans-acting factors secreted by nearby stroma to enhance ECM degradation.

**Fig 2:**
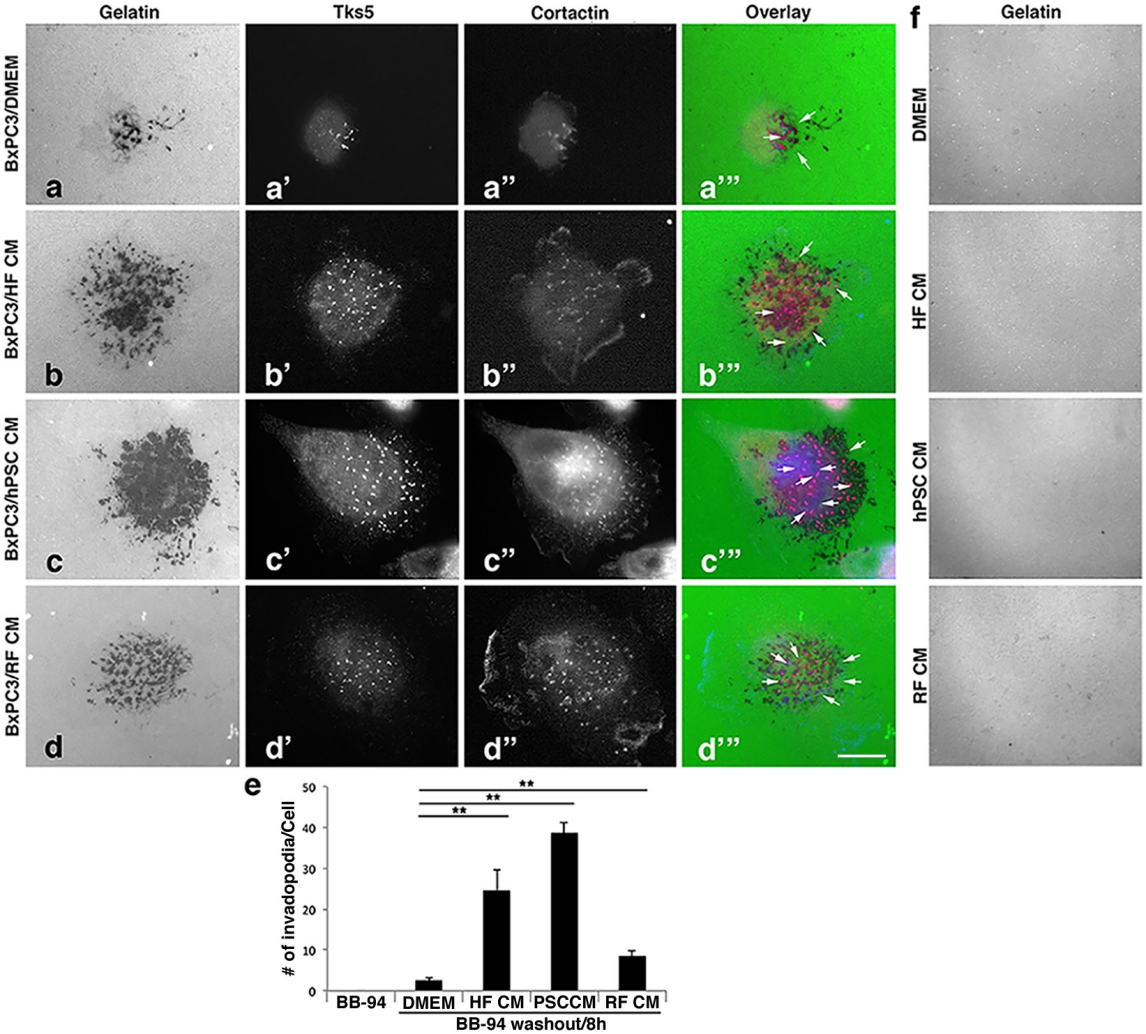
Conditioned medium from fibroblasts stimulates the accumulation of functional invadopodia in pancreatic cancer cells. The presence of CM from fibroblasts increases the number of invadopodia in PDAC cells. (a-d”) Representative images of BxPC3 cells seeded onto green fluorescent gelatin-coated coverslips and incubated with the MMP inhibitor BB-94 (2μM) overnight. (a-d) The inhibitors were removed by extensive washing and the cells were allowed to initiate gelatin degradation by incubation for 8 h with either DMEM alone (a, a’, a”, a”‘), CM collected from HFs (b, b’, b”, b”‘), hPSCs (c, c’, c”, c”‘), or RFs (d, d’, d”, d”‘). Cells were then fixed and stained with antibodies to the invadopodial marker Tks5 and cortactin. Scale bar = 10μm. (e) Quantification showing that CM from HFs, hPSCs, and RFs increased the number of invadopodia in BxPC3 cells markedly compared to the BB-94 and DMEM controls (≥10 cells per condition). Graphs represent averages ± SEM from 3 independent experiments. **p <0.01. (f) No degradation was observed when conditioned medium from the indicated cells was incubated with green fluorescent gelatin-coated coverslips for 8 hours.

To test if the number of invadopodia is increased by exposure to stromal-generated CM, BxPC3 cells were plated on green fluorescent gelatin-coated coverslips and stained for the invadopodial markers Tks5 and cortactin. Consistent with data shown in Fig 1j–1o, BxPC3 cells co-cultured with the CM from fibroblasts exhibited a substantial increase in matrix degradation (Fig 2a–2d). Importantly, these cells also displayed a marked increase in the number of both Tks5 and cortactin-positive invadopodia. A 3–10 fold increase in the number of invadopodia were observed in the cells co-cultured with CM from HFs (Fig 2b), hPSCs (Fig 2c), or RFs (Fig 2d) compared to the DMEM control treated cells (2a, e). Remarkably, the CM also induced a remarkable increase in invadopodia formation in the PANC-1 cells (S1k Fig), which do not normally form invadopodia. No functional invadopodia were detected in the presence of the MMP inhibitor BB-94 (Fig 2e), and the conditioned medium by itself did not lead to any degradation of the matrix (Fig 2f). These observations suggest that fibroblasts may secrete factors/proteins to stimulate invadopodia accumulation and function in pancreatic cancer cells while enhancing their matrix remodeling ability.

### Fibroblasts secrete MMP2 to enhance the invasive properties of pancreatic cancer cells

**Fig 3:**
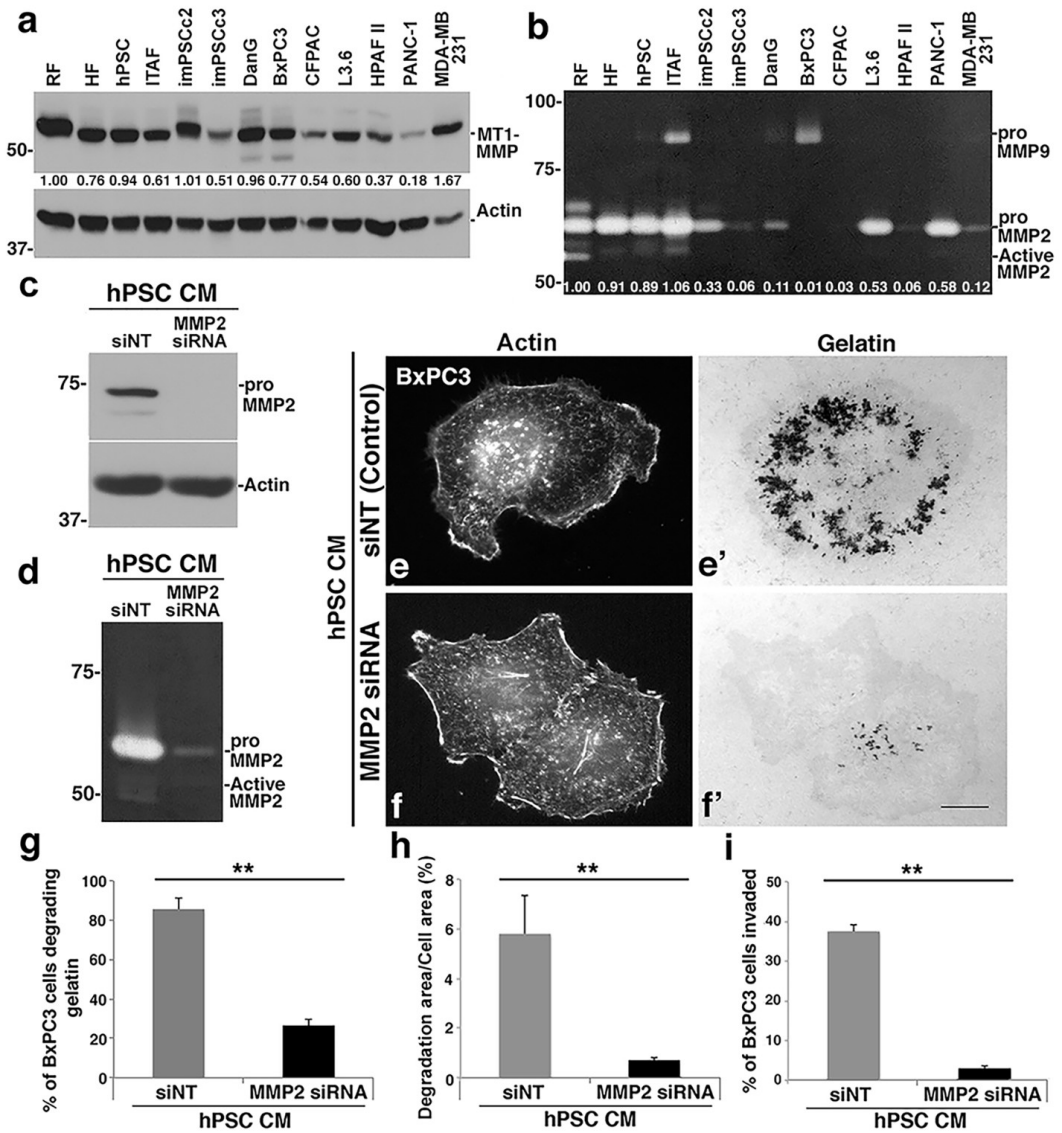
MMP2 in stromal cell conditioned medium is important to drive PDAC cell invasion. (a) Western blot analysis of MT1-MMP protein levels in 6 different PDAC cell lines, 1 breast cancer cell line (MDA-MB-231) and 6 different stromal cell lines. MT1-MMP levels were normalized to actin and compared to RF, and relative values are shown below each band. (b) Corresponding zymographic analysis of MMP2 and MMP9 levels in the same cells. Total MMP2 levels (pro+active) were quantified, normalized to RFs, and are shown below each band. (c-h) MMP2 depletion from fibroblast CM reduces matrix degradation by BxPC3 cells. (c) hPSC cells were treated with control siRNAs or siRNAs against MMP2 for 72 h. The knockdown efficiency of MMP2 in hPSC cells was confirmed by western blot. (d) The depletion of MMP2 in the CM collected from hPSC cells treated with siRNA against MMP2 was verified by zymography. (e) Fluorescence images of BxPC3 cells seeded on green fluorescent gelatin-coated coverslips and then cultured in CM collected from hPSC cells treated with control siRNA or siRNA against MMP2. Depletion of MMP2 in hPSC cells markedly reduced the matrix degradation by BxPC3 cells (f’) compared to the degradation in medium gathered from control cells (e’). The cell area was indicated by phalloidin staining for actin (e, f). Scale bar = 10μm. (g-h) Bar graphs depict quantification of gelatin degradation after 8 h. MMP2 depletion in hPSC CM results in a >3-fold decrease in the percent of BxPC3 cells degrading the matrix (≥100 cells per condition, g) and a >8-fold decrease in the area degraded per cell in BxPC3 cells (≥10 cells per condition, h). (i) The depletion of MMP2 in conditioned medium from hPSC cells inhibited BxPC3 cell transwell invasion. BxPC3 cells were seeded in transwell invasion assays for 6 h with CM from hPSC cells treated with control siRNA or siRNA against MMP2. The results represent the mean ± SEM of 3 independent experiments. *p <0.05. **p <0.01.

The data presented above indicate that stromal fibroblasts stimulate tumor cell invadopodial degradation through secretion of a soluble factor. We hypothesized that stromal cells might secrete MMPs that can cooperate with PDAC cells to accentuate ECM degradation. To test this prediction, we analyzed the proteases found in both stromal and tumor cells with a focus on the soluble MMP2 and MMP9 proteases and the transmembrane MT1-MMP proteases that are known to drive matrix degradation in many tumor types, including breast, prostate, and pancreatic cancer [11, 37–40]. The expression levels of these 3 proteases were compared using western blot and zymography (Fig 3a and 3b) in 6 different stromal cell lines (RF, HF, hPSC, ITAF, imPSCc2, imPSCc3), as well as 6 different PDAC cell lines (DanG, BxPC3, CFPAC, L3.6, HPAF-II, PANC-1) and 1 breast cancer cell line (MDA-MB-231). Within the stromal cells, RF and HF are normal fibroblasts while hPSC, ITAF, imPSCc2 and imPSCc3 are pancreatic stellate cells from either human or mice [41, 42]. Interestingly, the stromal cell lines, particularly RF, HF, hPSC, and ITAF cells, displayed relatively high levels of MMP2, especially pro-MMP2, in comparison to the tumor cells. Of all the cells we tested, MMP9 was detected in two cells lines (ITAF and BxPC3 cells). MT1-MMP on the other hand was ubiquitously expressed in PDAC and stromal cells.

Because of the elevated MMP2 expression observed in the stromal cells, we tested if this soluble protease might act as the trans-acting factor responsible for promoting PDAC cell matrix degradation. To this end, CM from the different stromal cells was added to BxPC3 cells seeded on fluorescent gelatin and analyzed for changes in ECM degradation. Correlative measurements were made to test if the stromal cells with the greatest MMP2 levels induced greater matrix degradation than that induced by cells with lower MMP2 expression/activity. Accordingly, BxPC3 cells exhibited a higher degradative response correlated with the MMP2 levels of the stromal cells (iTAF, imPSCc2, imPSCc3) from which the CM was derived (S3d–S3h Fig). Additional correlative studies using CM harvested from a variety of PDAC tumor cell lines with varying MMP2 levels (Fig 3b) were also performed (S3i Fig). Consistent with the observations made using stromal cell CM, the matrix degradation activity exhibited by BxPC3 cells was closely correlated to the MMP2 level depicted in Fig 3b. Thus, PDAC cell CM with the greatest MMP2, including PANC-1 and L3.6, conferred more robust matrix remodeling than tumor cells with low MMP2, including HPAF-II and CFPAC (S3j–S3q Fig). These findings are consistent with the concept that MMP2 from the CM is a key factor in stimulating PDAC cells, regardless of the cellular source of the CM.

In order to address the role of MMP2 within fibroblast CM more directly, hPSC and HF cells were treated for 72 h with control siRNA or siRNA to reduce the levels of MMP2, and we tested if CM from these cells had a reduced ability to stimulate PDAC cell degradation. A decrease in the MMP2 levels and activity in the CM from the transfected cells was confirmed by western blot and zymography (Fig 3c and 3d; S4a and S4b Fig). The MMP2-depleted stromal cell CM was then applied to cultured BxPC3 cells seeded on fluorescent gelatin-coated coverslips to assess matrix degradation. Strikingly, while CM from control-treated stromal cells stimulated BxPC3 matrix degradation, a marked reduction in degradation was observed in BxPC3 cells incubated with CM from stromal cells lacking MMP2. As shown in Fig 3e–3h and S4c–S4f Fig, MMP2-depleted CM resulted in a decrease in the number of PDAC cells exhibiting gelatin degradation by over 3 fold and a decrease in the area of degradation by over 8 fold.

To test if MMP2 secreted from stromal cells contributes to PDAC cell invasion *in vitro*, we employed the transwell invasion assays described above in Fig 1p. BxPC3 cells were seeded in the top chamber with CM from control-treated stromal cells or MMP2-depleted CM. The invasiveness of BxPC3 cells was analyzed after 8 h by counting the percentage of cells invading from the top to the bottom side of the chamber. Cells incubated with the CM from control cells exhibited 12-fold more invasion than did the cells presented with the MMP2-depleted CM (Fig 3i), consistent with the prediction that MMP2 from CM contributes to PDAC cell invasion.

As an additional approach to the siRNA-mediated MMP2 depletion studies described above, the MMP2/9 inhibitor SB-3CT was utilized to inhibit stromal cell MMP2 activity directly. To first verify the activity of this inhibitor, a dose-response analysis was performed by adding increasing concentrations of this inhibitor to CM collected from RFs (RF CM). As shown by the zymograph in S4g Fig, increasing the concentration of SB-3CT from 0 to 35nM resulted in a corresponding stepwise attenuation of MMP2 activity. Accordingly, vehicle alone or 28nM of SB-3CT was added to RFs prior to the collection of CM and incubation with BxPC3 cells, and the effects on degradation were recorded (S4h–S4i’ Fig). In support of the MMP2 siRNA knockdown experiments, the inhibition of MMP2 by the SB-3CT protease inhibitor resulted in a 3-fold decrease in the percentage of BxPC3 cells degrading matrix and a 30-fold reduction in the degradation area per BxPC3 cell area (S4j and S4k Fig). Taken together, these data provide strong evidence for the action of stromal cell MMP2 as an important contributor to PDAC cell invasion.

### The importance of PDAC cell MT1-MMP in matrix remodeling

**Fig 4:**
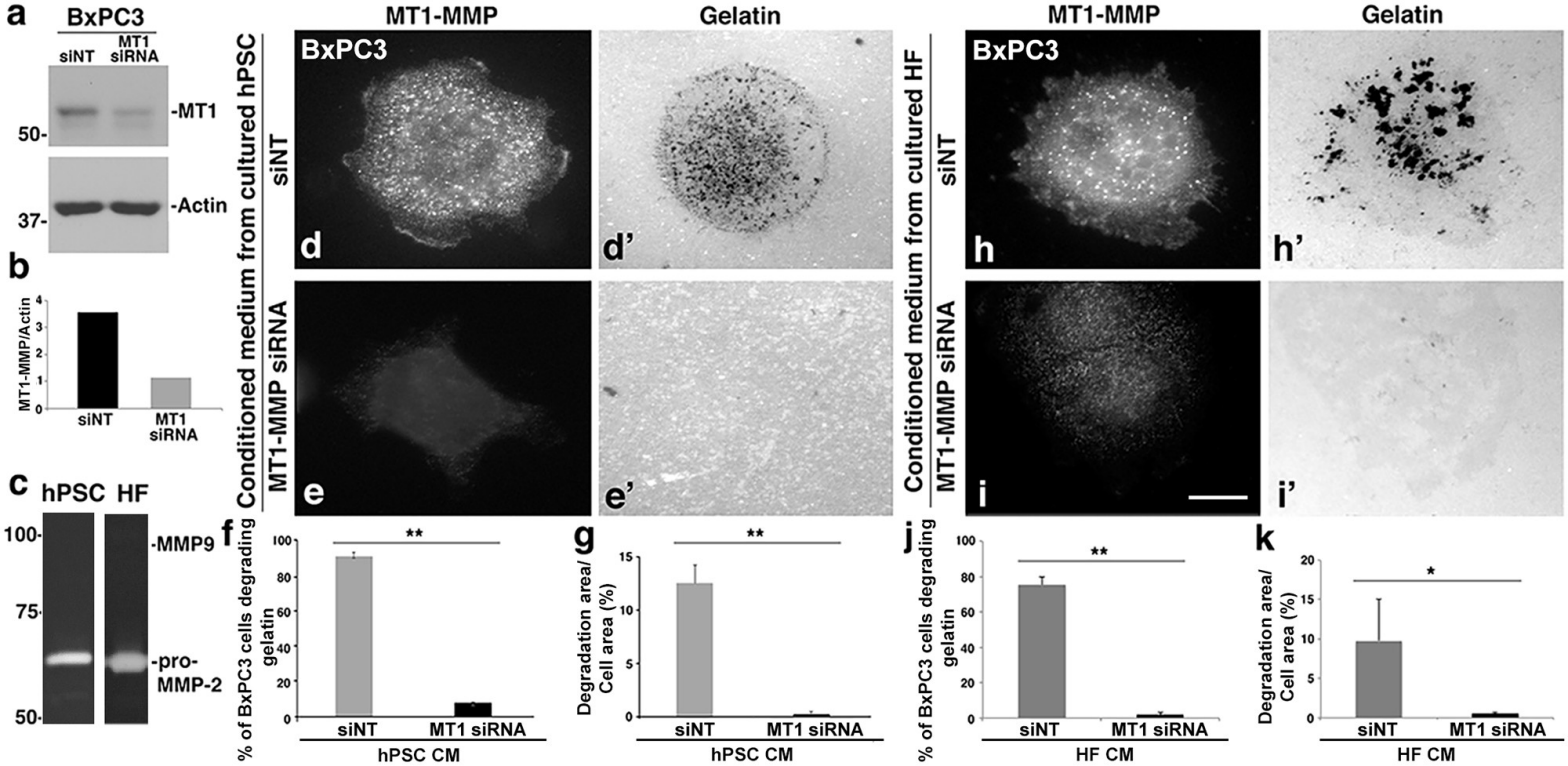
PDAC cell MT1-MMP is essential for PDAC cell ECM remodeling. MT1-MMP depletion in BxPC3 cells inhibits matrix degradation in the presence of fibroblast CM. (a) Western blot of BxPC3 cells treated for 72 h with control or siRNA to MT1-MMP. The relative levels of MT1-MMP normalized to actin are depicted in the bar graph (b). (c) Zymogram of CM collected from hPSCs or HFs confirming the presence of pro-MMP2 in these cells. (d-e, h-i) Fluorescence micrographs of BxPC3 cells treated with control siRNAs or MT1-MMP-targeted siRNAs for 3 days. The cells were then seeded onto green fluorescent gelatin-coated coverslips and cultured in CM from hPSCs (d-e) or HFs (h-i) for 8 h prior to fixation and staining with antibodies to MT1-MMP. The knockdown of MT1-MMP in the BxPC3 cells significantly reduced ECM degradation by these cells in the presence of CM from both hPSCs and HFs. Scale bar = 10μm. (f-g, j-k) Bar graphs quantifying matrix degradation by BxPC3 cells as described above. Both the percentage of BxPC3 cells degrading matrix (f, j) and the total area of matrix degraded per cell (g, k) in BxPC3 cells decreased dramatically following MT1-MMP depletion in PDAC cells incubated with CM from hPSCs (f, g) or HFs (j, k). Graphs represent averages ± SEM from at least 3 independent experiments. *p <0.05. **p <0.01.

MMP2 is initially secreted as a pro-form and becomes activated outside the cell via the action of the MT1-MMP residing on the plasma membrane [37]. Based on the protease western blotting and zymography in Fig 3a and 3b, we predicted that MMP2 secreted by the stromal fibroblasts is cleaved and activated by MT1-MMP on the PDAC tumor cells. To test this premise, BxPC3 (Fig 4) or DanG (S5a–S5f Fig) cells were plated on fluorescent gelatin-coated coverslips after transfection with control siRNA or MT1-MMP-targeted siRNA. These cells were then incubated with either hPSC or HF CM which have high amounts of MMP2 (Figs 3b and 4c; S5b Fig) for 8 h to allow matrix degradation before fixation. The expression levels of MT1-MMP in individual PDAC cells were monitored by western blot and immunofluorescence (IF) staining. As displayed in Fig 4 and S5 Fig, MT1-MMP depletion in BxPC3 cells completely abolished the increased matrix degradation stimulated by hPSC CM (Fig 4d–4g) or HF CM (Fig 4h–4k). Similar results were observed in DanG PDAC cells depleted of MT1-MMP and treated with HF CM (S5a–S5f Fig). These data suggest that MT1-MMP provided by the PDAC tumor cells, and not the stromal cells, drives MMP2-dependent matrix degradation.

Conversely, we tested if overexpression of MT1-MMP was sufficient to induce matrix degradation in PANC-1 cells, which do not degrade gelatin. PANC-1 express pro-MMP2, but have very low levels of MT1-MMP (Fig 3a and 3b). Consistent with our prior studies, overexpression of MT1-MMP in PANC-1 induced a dramatic increase in matrix degradation. To test if this degradation required the pro-MMP2 secreted by PANC-1, we depleted MMP2 expression in PANC-1 cells using siRNA. Indeed, knockdown of MMP2 blunted the matrix degradation induced by overexpression of MT1-MMP, supportive of cross-talk between these two pro-invasive proteinases in pancreatic tumor cells (S5g–S5m Fig).

**Fig 5:**
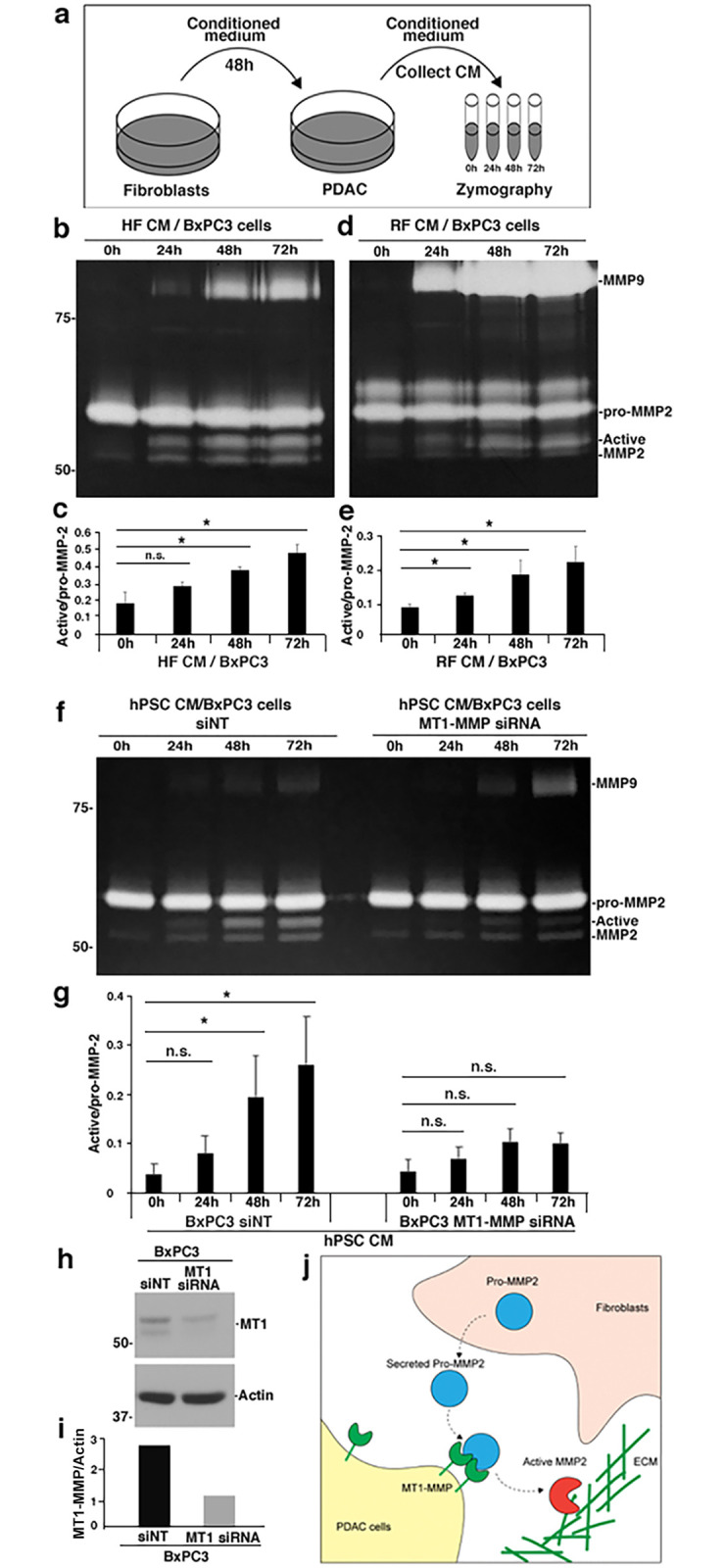
MMP2 secreted from stromal cells is activated by PDAC cell MT1-MMP. (a) An illustration of the experimental design followed for this experimental approach. CM from HFs, RFs, or hPSCs was collected and applied to BxPC3 cells. Subsequently, CM from the PDAC cell cultures was collected after 0, 24, 48 or 72 h and subjected to zymography. (b,d) Representative zymograms showing MMP2 activity of the CM described above. The activity of this protease from the HF CM (b) or RF CM (d) was increased by longer incubation times with BxPC3 cells. (c, e) Quantitative graphs depicting the ratio of pro/active MMP2 at each timepoint, demonstrating the increase in activity of MMP2 from the zymograms of at least 3 independent experiments. (f) MT1-MMP was depleted in BxPC3 cells by siRNA transfection. MMP2 in CM from hPSCs is also activated over time when incubated with control BxPC3 cells, but this is inhibited following MT1-MMP knockdown. (g) Quantitation of the ratio of pro/active MMP2 at each timepoint from zymograms from 3 independent experiments. Averages ± SEM. *p <0.05. **p <0.01. n.s, not statistically significant. (h-i) The knockdown of MT1-MMP was verified by western blot and quantified by normalizing MT1-MMP to actin. (j) Proposed model showing the functional relationship between tumor cell MT1-MMP and stromal cell MMP2 during matrix remodeling. MT1-MMP presented at the surface of PDAC tumor cells can cleave and activate the pro-MMP2 provided by fibroblasts. The result of this activation is enhanced matrix remodeling and increased cancer cell invasion.

To pursue this concept further, zymography was employed to test if the MMP2 derived from the stromal cells was activated by the tumor cells. To this end, CM from hPSCs, HFs, or RFs was collected and applied to BxPC3 or CFPAC PDAC cells for incubation for 0, 24, 48, or 72 h. Subsequently, CM from the PDAC cells was collected for zymography as depicted in Fig 5a. As predicted, a steady increase in the active form of MMP2 was observed over increasing co-incubations (Fig 5b–5g; S6a–S6d Fig). This finding suggests that MMP2 secreted by stromal cells can be activated by tumor cells. To confirm the requirement for tumor cell MT1-MMP in this process, we used siRNA to deplete MT1-MMP in BxPC3 tumor cells. Importantly, knockdown of MT1-MMP blocked the activation of MMP2 upon incubation with BxPC3 cells, demonstrating that MT1-MMP is crucial for this activation (Fig 5f and 5g). Notably, we have not observed any active MMP2 secreted by BxPC3 or CFPAC cells by testing their conditioned medium collected at 24h, 48h, or 72h (S6e Fig). These data support the concept that the stimulation of pancreatic cancer cell matrix degradation by fibroblast-secreted MMP2 requires the activation by MT1-MMP residing on the tumor cells. Thus, crosstalk between fibroblasts and cancer cells appears critical for promoting tumor stromal remodeling, as illustrated in Fig 5j.

## Discussion

### The tumor microenvironment in cancer metastasis

In this study we have focused on the interactive contributions of stromal and PDAC tumor cells to pro-invasive matrix remodeling. The specific tumor microenvironment for pancreatic cancer has two major features that include fibrotic desmoplasia as well as an immunosuppressive component [26, 43]. Here we focus on desmoplasia where PSCs are a central contributing factor [20]. PSCs are resident mesenchymal cells located in the pancreas. Quiescent PSCs store lipid droplets, express MMPs including MMP2, MMP9 and MMP13, as well as MMP inhibitors, and maintain normal tissue homeostasis [32]. Upon activation by growth factors and cytokines that are secreted by immune cells, pancreatic cancer cells, and endothelial cells, PSCs can differentiate into cancer-associated fibroblasts (CAFs), leading to a loss of lipid droplets with a concomitant upregulation of MMPs and ECM components [44]. Importantly, PSCs can differentiate into different subtypes of CAFs, including those adjacent to neoplastic cells expressing high levels of α-smooth muscle actin (α-SMA), while others may secrete IL6 and other inflammatory mediators without SMA expression [20, 21, 26, 45]. Activated CAFs can then promote metastasis through the production and remodeling of ECM, the secretion of growth factors and cytokines that promote cancer cell survival and invasion, as well as angiogenesis [46]. Thus, the biology of the supportive PDAC stroma is complex and critical for invasion and metastasis. Accordingly, this study utilized both PSCs and normal fibroblasts to cast a wide net in an attempt to test for conserved interactive processes between these stromal cells and PDAC tumor cell lines, and determine how these processes contribute to cancer cell invasion.

Through a combination of the cell models described above, we report a mechanism of matrix metalloprotease activation in PDAC through a crosstalk between the tumor cells themselves and the adjacent stroma (Fig 1). Importantly, this invasion-promoting cross communication appears to be mediated by the synergistic interaction of membrane-bound MT1-MMP residing on tumor cells with the soluble MMP2 secreted by PSCs and normal fibroblasts (Fig 3). As normal human and rat fibroblasts similarly provided MMP2 to promote matrix degradation and tumor cell invasion, these data suggest that tumor cell invasion could be supported by the stroma even prior to the conversion to CAFs, for example, early in tumor development, or as invading tumor cells interact with normal fibroblasts in other tissues. Interestingly, we observed that different populations of PDAC tumor cells can also activate other epithelial PDAC tumor cells to become more invasive (S3i–S3q Fig). As PDAC can have significant intratumoral heterogeneity, this finding suggests that cross-talk among tumor cells with complementary MMP expression may also promote invasion [47].

### Trans-activation of MMP2 by MT1-MMP

From the findings presented here it appears that MMP2 and MT1-MMP are directly involved in the degradation of extracellular matrix components. It has been reported that MMP2 activation by MT1-MMP contributes to pancreatic cancer progression and invasion, as both fibroblasts and cancer cells can express these proteases [48]. However, the specific contributions and actions of the stroma versus epithelial tumor cells in protease-based ECM degradation are undefined. In this study, a comparison of MMP2 and MT1-MMP expression in 6 stromal cells and 7 tumor cell lines revealed a strong correlation between the levels of protease expression and the capacity of these cells to degrade gelatin. We observed that MMP2 is highly expressed and secreted by fibroblasts and PSCs, whereas MT1-MMP is expressed in both fibroblasts and cancer cells (Fig 3 and 3b).

Surprisingly, MMP2 from stromal fibroblasts appears to be largely inactive, despite the co-expression of MT1-MMP by these cells (Fig 3a and 3b and S3i Fig). Importantly, this indicates that these stromal cells have only a modest capacity for self-activation of their own MMP2 and require a trans-activation of this protease by the MT1-MMP expressed by the nearby tumor cells (Fig 5 and S6 Fig). Reciprocal trans-activation of tumor cell matrix remodeling by the nearby stroma is also demonstrated by the fact that PANC-1 cells, epithelial tumor cells that exhibit very modest matrix degradation, can be activated to increase matrix remodeling via incubation with media from MMP2-secreting stromal cells, even though PANC-1 also express pro-MMP2 (S1 Fig). Similarly, other pancreatic cancer cells such as DanG, HPAF-II, L3.6, and BxPC3 cells all express various levels of both MMP2 and MT1-MMP (Fig 3a and 3b). However, similar to fibroblasts they appear incapable of self-activating MMP2 as minimal active MMP2 is detected by zymography (Fig 3b and S6e Fig). Thus, trans-activation of MMP from one cell type depends upon the activity of MT1-MMP from another. These findings further support the premise that MMP2 activation is a trans-acting event requiring the participation of both cell populations.

It remains unclear why MT1-MMP based activation of secreted MMP2 does not occur in a *cis*-fashion at the surface of tumor or stromal cells that generates both proteases. Perhaps additional cofactors or the topological orientation of these two proteases play a key role. It is noteworthy that the soluble protease MMP9, while not expressed in the majority of the pancreatic cancer cells and stromal cells tested here, has been implicated in PDAC progression as well (Fig 3b) [10, 38]. While there are undoubtedly additional factors mediating cross-talk between fibroblasts and tumor cells, which may regulate invadopodial dynamics, migration, protein expression, proliferation, and survival, our data indicate the requirement for the MT1-MMP/MMP2 tumor/stromal cell axis for pro-invasive matrix degradation. The findings reported here underscore the need for further understanding of this complex crosstalk between the cell types of this lethal tumor. They also emphasize the importance of defining the biology of this stroma that drives the desmoplastic nature of PDAC and may represent over 80% of PDAC tumor mass. Such understanding will prove helpful toward developing new therapies toward attenuating the metastatic invasion of this lethal cancer.

## Methods

### Cell culture

BxPC-3 cells (ATCC CRL-1687) and L3.6 (Provided by Dr. Isaiah J. Fidler, University of Texas, Houston, TX) were maintained in RPMI-1640 medium with 10% fetal bovine serum (FBS) (Sigma, St. Louis, MN), 100 U/ml penicillin (P), and 100 mg/ml streptomycin (S) (Life Technologies, Carlsbad, CA). The following cell lines were maintained in DMEM medium with 10% FBS and 1% P/S: Rat fibroblasts (RF, ATCC CRL-1213), Human foreskin fibroblasts (HF, ATCC CRL-110), DanG (Provided by Dr. Martin Fernandez-Zapico, Mayo Clinic, Rochester, MN), PANC-1 (ATCC CRL-1469), and MDA-MB-231 (ATCC HTB-26), CFPAC-1 (ATCC CRL-1918). HPAF-II (ATCC CRL-1997) was maintained in MEM containing 10% FBS and 1% P/S. hPSC was from ScienCell (#3830, Carlsbad, CA, USA) and maintained in stellate cell medium (SteCM) as described previously [22]. A patient-derived PSC cell line, ITAF (hCAF), and two C57BL/6 mouse-derived immortalized PSC cell lines, imPSC c2 and imPSC c3, were provided by Dr. Raul Urrutia, Medical College of Wisconsin and maintained as described previously [41, 42]. BxPC3 (GFP) is pEGFP N1 vector stable expressed in BxPC3 and maintained with G418 sulfate (400 μg/ml, CORNING, Mediatech Manassas,VA) in RPMI1640 containing 10% FBS and 1% P/S. All cells were grown in a 5% CO_2_ incubator at 37°C.

For co-culture experiments, fibroblasts:cancer cells were plated in the following ratios: human fibroblasts 7:1, hPSCs 5:1, rat fibroblasts 3:1. To generate conditioned medium, cells were cultured at confluence for 24–48 hours in the absence of serum. Cells incubated with conditioned medium were compared to cells in serum-free medium.

The following inhibitors were used as indicated: MMP inhibitor BB-94 (2 μM, Tocris Bioscience, Bristol, UK), MMP-2/MMP-9 inhibitor SB-3CT (28 nM, Sigma, St. Louis, MO, USA).

Cells were transfected with siRNA using Lipofectamine RNAiMAX (Life Technologies) according to the manufacturer’s instructions. Nontargeting siRNA (#1, Catalog #D-001810-01, siNT) and all targeting siRNAs were from Dharmacon (GE Healthcare, Lafayette, CO, USA): Human MMP-14 (MT1-MMP, #D-004145-02-0010); Human MMP-2 (5′-GGAGAGCUGCAACCUGUUU-3′) [49].

### Immunoblotting

To prepare cell homogenates for western blotting, cells were lysed in NP-40 lysis buffer (20 mM Tris-Cl, pH 8.0, 137 mM NaCl, 10% glycerol, 1% NP-40, 2 mM EDTA) containing complete protease inhibitors (Roche) followed by sonication. A BCA assay (Pierce) was used to determine the protein concentrations. Equal amounts of proteins (20–40 μg) were resolved by SDS-PAGE then transferred to PVDF membranes for antibody probing. Primary antibodies used included MT1-MMP (Abcam), MMP2 (EP1183Y, Abcam, Cambridge, MA), Actin (Sigma) [22, 50], and Tks5 (Millipore-Sigma, Milwaukee, WI). Horseradish peroxidase-conjugated secondary antibody was purchased from Biosource International. SuperSignal West Pico Chemiluminescent substrate (Thermo Fisher Scientific) was used to detect immunoreactive signals with autoradiographic films (HyBlot CL). Films were scanned by a CanoScan LiDE120 scanner (Canon). Bands were quantified by the area density measurement using Chemi Doc-IT^2^ Imager software (UVP).

### Gelatin zymography

Conditioned medium from cultured cells was collected as previously described [22]. Briefly, cells were cultured in medium without serum for 24–48 hours at similar confluency. Upon collection, the culture medium was clarified by centrifugation at 6000rpm for 2 min. Then the conditioned medium was mixed with zymogram sample buffer (BioRad, Hercules, CA) and incubated at room temperature for 10 minutes. The conditioned medium was resolved on 7.5% SDS-PAGE gels containing 1 mg/ml gelatin. After electrophoresis, the SDS-PAGE gel was then transferred into 2.5% Triton X-100 and incubated at room temperature for 40 minutes with shaking. After incubation, the gel was rinsed with incubation buffer (50 mM Tris-B pH8.0; 150 mM NaCl; 10 mM CaCl; 0.05% NaN_3_) before being soaked in incubation buffer at 37°C for 20~24 hours. After incubation, the gel was rinsed with dH_2_O 3 times before staining with Coomassie blue for 40 minutes at room temperature. Finally, destaining was performed at room temperature for 2 hours before the gels were imaged. Active and Pro-MMP2 bands were quantified by the area density measurement using Chemi Doc-IT^2^ Imager software (UVP).

### Immunofluorescence

Cells were fixed as described previously [22]. TRITC-phalloidin and Phalloidin-Atto 390 (Sigma Aldrich) were used to visualize Actin. The coverslips were mounted with Prolong mounting medium (Life Technologies) before imaging. Fluorescence images were acquired using epifluorescence microscopes (Axio Observer and Axiovert 200; Carl Zeiss MicroImaging) using a 63x oil objective with iVision software or Zen software. Adobe Photoshop software (Adobe) was applied to process and adjust the images uniformly.

### Matrix degradation assays

Gelatin-coated coverslips were prepared using 0.2% gelatin (Sigma Aldrich) and diluted Oregon Green-conjugated gelatin (Invitrogen) and Cy3-conjugated gelatin (EMD Millipore), as previously described [50, 51]. Cells were seeded on the gelatin-coated coverslips in the presence of the MMP inhibitor BB-94 overnight (2μM) and in the presence of 10% FBS. The inhibitor was washed out the following day to allow matrix degradation by MMPs. The cells were incubated in the appropriate serum-free medium for the indicated time before fixation. After TRITC-phalloidin staining (Sigma Aldrich) or immunofluorescence for the invadopodial markers Tks5 (Millipore-Sigma), and cortactin (4F11, Millipore-Sigma) the cells were imaged with an AxioObserver D.1 epifluorescence microscope (Carl Zeiss, Thornwood, NY, USA). The percentage of cells degrading the matrix was determined with at least 100 randomly imaged cells. The degradation area per cell area was quantified with at least 10 cells per condition with ImageJ software, and was normalized to the cell area [52]. The number of invadopodia per cell was quantified manually using ImageJ and Adobe Photoshop by counting the number of spots that were positive for cortactin/Tks5 staining and gelatin degradation (Fig 2), or actin staining and gelatin degradation (S1k Fig).

### Transwell assays

24-well transwell chambers containing 8-micron diameter pores (Millipore) were coated with 0.3% gelatin prior to the seeding of cells. 2x10^4^ BxPC3 cells cultured in the indicated conditioned medium were seeded on the top of the transwell. The bottom of the culture dish was filled with culture medium containing 10% FBS to promote invasion over 6 h. After 6 h, the cells on the top and bottom of the permeable membrane were fixed and stained with DAPI. Cell invasiveness was determined by measuring the number of DAPI-positive nuclei at the top versus the bottom of the membrane.

#### Statistical analysis

Data were analyzed using Microsoft Excel, and are represented as the mean +/- standard error. A two-tailed unpaired student’s t-test was used to calculate statistical significance, with p<0.05 indicating a statistically significant difference.

## Supporting information

S1 Fig Fibroblasts stimulate the invasive properties of PANC-1 cells. (a-d) The physical presence of fibroblasts dramatically promotes ECM degradation by PANC-1 cells. Fluorescence images of PANC-1 cells seeded onto green fluorescent gelatin-coated coverslips (a, a’) or co-cultured with stromal hPSC cells (b, b’), showing an increase in gelatin degradation over 16 h induced by the presence of the hPSCs. Scale bar = 10μm. (c-d) Bar graphs showing quantification of gelatin degradation. Co-culture of PANC-1 cells with the stromal cells induce the ability of PANC-1 cells to degrade matrix, in both the number of cells exhibiting gelatin degradation (≥100 cells per condition, c), and the area of degradation per cell (≥10 cells per condition, d). Stars indicate the ECM degradation caused by PANC-1 cells. Scale bar = 10μm. Graphs represent averages ± SEM from 3 independent experiments. **p <0.01. (e-j) CM from fibroblasts promotes ECM degradation by PANC-1 cells. PANC-1 cells were seeded onto green fluorescent gelatin-coated coverslips in DMEM only (e) or CM collected from HFs (f), hPSCs (g), or RFs (h), and matrix degradation was quantified after 8 h. In the presence of CM from stroma, PANC-1 cells start degrading the gelatin substrate, quantified both with the percentage of cells degrading matrix (≥100 cells per condition (i) and the area degraded per cell in PANC-1 cells (≥10 cells per condition (j), compared to the DMEM control. (k) Invadopodia were scored by quantifying actin puncta that colocalized with regions of matrix degradation. Scale bar = 10μm. Graphs represent averages ± SEM from 3 independent experiments. *p <0.05. **p <0.01.
(TIF)

S2 Fig Conditioned media from RFs stimulates the ECM remodeling ability of pancreatic cancer cells. (a-h) Representative fluorescence images showing that CM from RFs dramatically enhanced gelatin degradation by DanG (a-b), HPAF-II (c-d), CFPAC (e-f), and MBA-MD-231 (g-h) cells over an 8 h period compared to the corresponding DMEM controls. Scale bar = 10μm. (i-j) Bar graphs showing quantification of gelatin degradation by the various PDAC tumor cells listed above. Quantification shows a marked increase both in the percent of PDAC cells degrading the gelatin matrix (≥100 cells per condition, i) and the degradation area per cell area (≥10 cells per condition, j). Graph represents averages ± SEM from 3 independent experiments. *p <0.05. **p <0.01. n.s, not statistically significant.
(TIF)

S3 Fig The ECM remodeling capacity of pancreatic cancer cells correlates with the level of MMP2 secreted into the culture media by stromal cells. (a-h) Fluorescence images of BxPC3 tumor cells seeded on green fluorescent gelatin-coated coverslips and cultured with (a) DMEM containing 10% FBS for 8 h before fixation, (b) the MMP inhibitor BB-94 (2 μM) for 8 h, (c-f) CM from different stromal cells for 8 h after BB-94 washout. (g,h) Graphs depicting matrix degradation by cells described above and quantified as either the percentage of BxPC3 cells degrading matrix (≥100 cells per condition, g) or the degradation area per cell area (≥10 cells per condition, h). These data suggest a direct correlation between the level of MMP2 in the stromal cell CM and the matrix degradation induced by the addition of this CM to the BxPC3 cells. Scale bar = 10μm. Bar graph represents averages ± SEM from 3 independent experiments. **p <0.01. n.s, not statistically significant. (i-q) MMP2 levels in CM collected from cancer cells correlates with the capacity to promote matrix degradation in recipient BxPC3 cells. (i) Zymography demonstrating MMP2 activity in 5 different PDAC cell lines compared to HFs. (j-o) Representative images of BxPC3 degrading matrix in response to CM derived from other tumor cells represented in the above zymogram. Note that the tumor cells secreting the lower amounts of MMP2 into the CM induce the least degradation by the recipient BxPC3 cells. Scale bar = 10μm. (p,q) Bar graph quantifying BxPC3 matrix degradation in response to CM from the distinct tumor cells. The percentage of BxPC3 cells degrading matrix was determined with ≥100 cells per condition and the degradation area per cell area was quantified with ≥10 cells per condition. Graphs represent averages ± SEM from 3 independent experiments.
(TIF)

S4 Fig The depletion of MMP2 from stromal cell conditioned medium reduces the ECM remodeling capacity of BxPC3 cells. (a) Western blot of HF cells treated with control siRNAs or siRNAs to reduce MMP2 levels. (b) Zymogram showing loss of MMP2 in the siRNA-treated cells described in (a). (c-d”) Fluorescence images of BxPC3 cells plated on green fluorescent gelatin-coated coverslips and incubated 8 h with CM collected from HFs cells treated with control siRNAs or siRNAs to reduce MMP2 levels. Phalloidin staining of actin was used to show cell borders (c, d). Scale bar = 10μm. (e,f) Quantitation of the experiment described above. Depletion of MMP2 from the HF-CM reduced the number of BxPC3 cells degrading matrix (≥100 cells per condition) and the total area of degraded substrate per cell compared to controls (≥10 cells per condition). (g) Zymogram showing a dose curve of the MMP2 inhibitor SB-3CT, when added to RFs, on MMP2 for 24 h prior to CM collection. (h,h’-i, i’) Fluorescence images of BxPC3 cells seeded on green fluorescent gelatin-coated coverslips and cultured in RF-CM with or without 28 nM of the SB-3CT inhibitor added for 8 h. (j,k) The MMP2 inhibitor reduced the percent of cells degrading matrix by 3 fold (≥100 cells per condition, j), and the area degraded per cell in BxPC3 cells by 30 fold (≥10 cells per condition, k). Graphs represent averages ± SEM from at least 3 independent experiments. *p<0.05, **p <0.01.
(TIF)

S5 Fig Depletion of MT1-MMP in DanG cells disrupts matrix degradation. MT1-MMP knockdown in DanG cells inhibits matrix degradation in the presence of HF CM. (a) DanG cells were transfected with control siRNAs or siRNA targeting MT1-MMP. Knockdowns were confirmed by western blotting. (b) The presence of MMP2 in HF CM was tested by zymography. (c-d”) Fluorescence micrographs of DanG cells that were transfected with control or siRNAs against MT1-MMP for 3 days and then seeded onto green fluorescent gelatin-coated coverslips and cultured in CM from HF cells for 8 h. The level of MT1-MMP in individual cells was tested by immunofluorescence using antibodies against MT1-MMP. Scale bar = 10μm. (e-f) Matrix degradation was quantified, showing that MT1-MMP depletion led to a substantial decrease both in the percent of cells degrading matrix (≥100 cells per condition, e) and in the area degraded per cell in DanG cells (≥10 cells per condition, f). (g) PANC-1 cells were transfected with control siRNAs or siRNA targeting MMP2. Knockdown was confirmed by western blotting. (h-k) Control or MMP2-depleted PANC-1 cells were transfected with mCherry vector (h,j) or mCherry MT1-MMP (i,k) and plated on fluorescent gelatin-coated coverslips. Matrix degradation was quantified, showing that MT1-MMP overexpression led to a substantial increase both in the percent of cells degrading matrix (>100 cells per condition, l) and the area of matrix degradation (> 10 cells per condition, m), which was partially suppressed by knockdown of MMP2. Graphs represent averages ± SEM from at least 3 independent experiments. *p <0.05. **p <0.01.
(TIF)

S6 Fig PDAC tumor cells activate MMP2 secreted from stroma. (a-d) CM from HFs or RFs was collected and applied to CFPAC cells. The CM from CFPAC cells were then further collected after 0, 24, 48 or 72 h and subjected to zymography. (a, c) Representative zymograms showing MMP2 activity of the CM described above. The amount of active MMP2 from the HF-CM (a) or RF-CM (c) was increased with longer incubation times with CFPAC cells. (b, d) Quantitation of the ratio of active to pro-MMP2 from the zymograms of 3 independent experiments. Data represent averages ± SEM. *p <0.05. **p <0.01. n.s, not statistically significant. (e) CM from BxPC3 cells and CFPAC cells were collected at 24, 48 or 72 h and subjected to zymography.
(TIF)

S1 Raw images (PDF)
