# Supplementary figures and images for: Synergistic metalloproteinase-based remodeling of matrix by pancreatic tumor and stromal cells
Source: PLoS One. 2021 Mar 19;16(3):e0248111. doi: 10.1371/journal.pone.0248111 (PMC7978280; doi:10.1371/journal.pone.0248111)

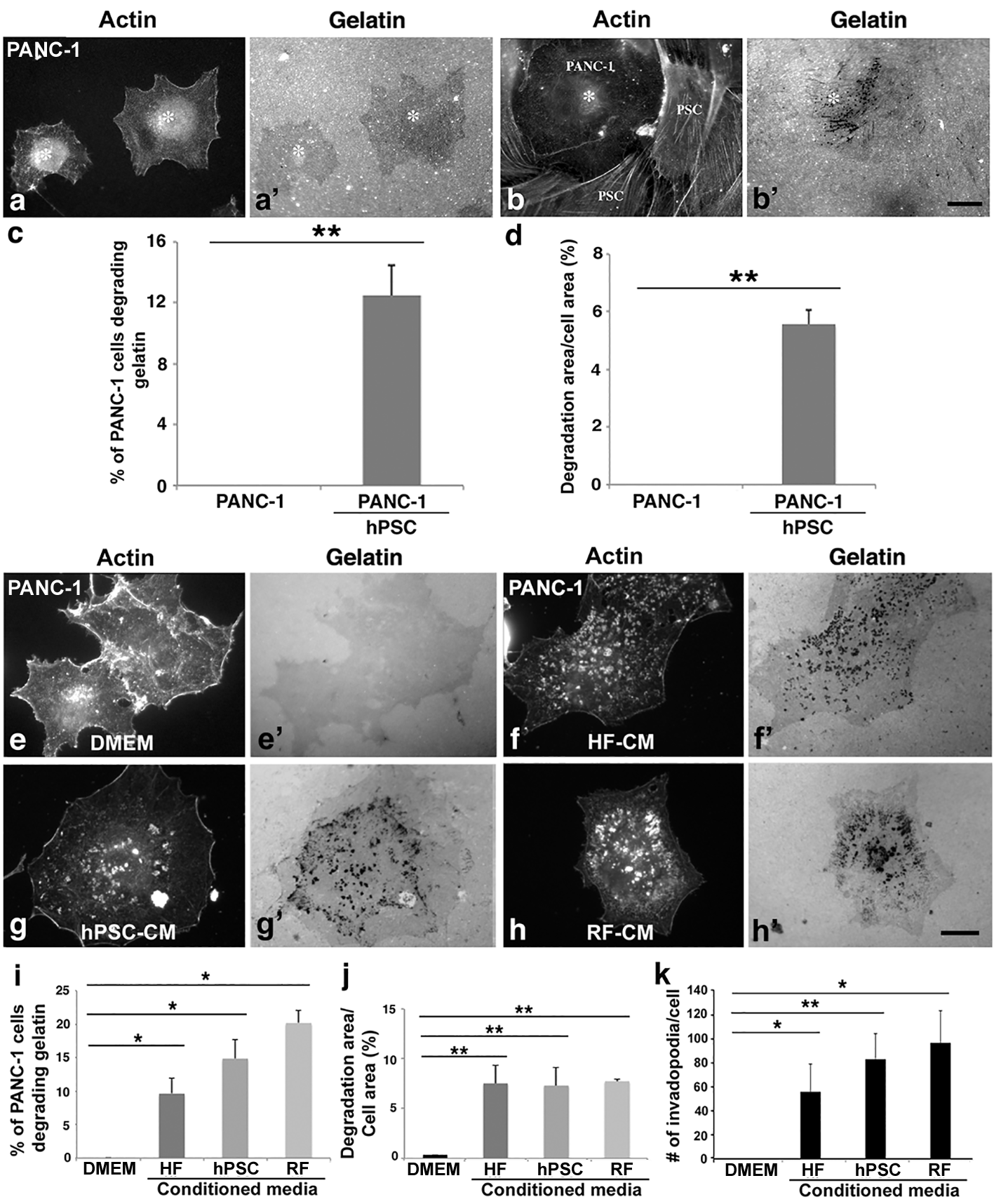

Supplement: S1 Fig — (a-d) The physical presence of fibroblasts dramatically promotes ECM degradation by PANC-1 cells. Fluorescence images of PANC-1 cells seeded onto green fluorescent gelatin-coated coverslips (a, a’) or co-cultured with stromal hPSC cells (b, b’), showing an increase in gelatin degradation over 16 h induced by the presence of the hPSCs. Scale bar = 10μm. (c-d) Bar graphs showing quantification of gelatin degradation. Co-culture of PANC-1 cells with the stromal cells induce the ability of PANC-1 cells to degrade matrix, in both the number of cells exhibiting gelatin degradation (≥100 cells per condition, c), and the area of degradation per cell (≥10 cells per condition, d). Stars indicate the ECM degradation caused by PANC-1 cells. Scale bar = 10μm. Graphs represent averages ± SEM from 3 independent experiments. **p <0.01. (e-j) CM from fibroblasts promotes ECM degradation by PANC-1 cells. PANC-1 cells were seeded onto green fluorescent gelatin-coated coverslips in DMEM only (e) or CM collected from HFs (f), hPSCs (g), or RFs (h), and matrix degradation was quantified after 8 h. In the presence of CM from stroma, PANC-1 cells start degrading the gelatin substrate, quantified both with the percentage of cells degrading matrix (≥100 cells per condition (i) and the area degraded per cell in PANC-1 cells (≥10 cells per condition (j), compared to the DMEM control. (k) Invadopodia were scored by quantifying actin puncta that colocalized with regions of matrix degradation. Scale bar = 10μm. Graphs represent averages ± SEM from 3 independent experiments. *p <0.05. **p <0.01. (TIF) [file pone.0248111.s001.tif]

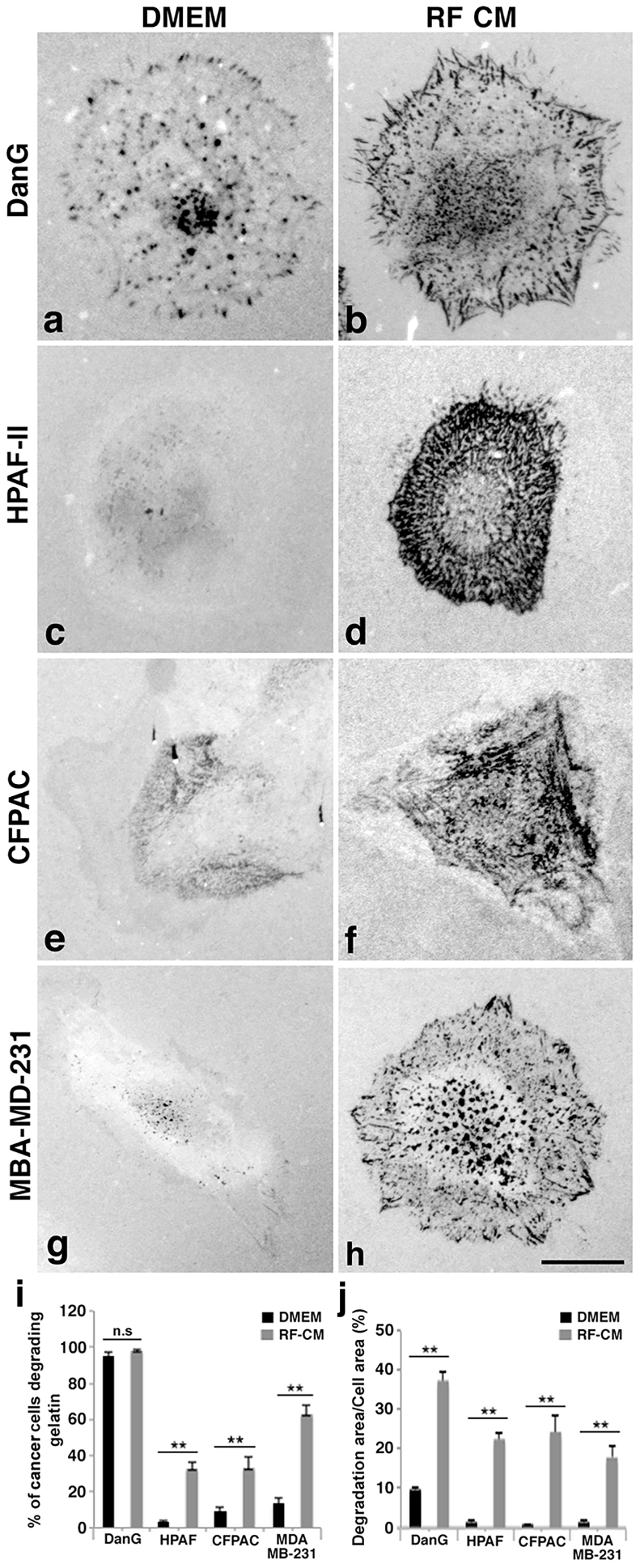

Supplement: S2 Fig — (a-h) Representative fluorescence images showing that CM from RFs dramatically enhanced gelatin degradation by DanG (a-b), HPAF-II (c-d), CFPAC (e-f), and MBA-MD-231 (g-h) cells over an 8 h period compared to the corresponding DMEM controls. Scale bar = 10μm. (i-j) Bar graphs showing quantification of gelatin degradation by the various PDAC tumor cells listed above. Quantification shows a marked increase both in the percent of PDAC cells degrading the gelatin matrix (≥100 cells per condition, i) and the degradation area per cell area (≥10 cells per condition, j). Graph represents averages ± SEM from 3 independent experiments. *p <0.05. **p <0.01. n.s, not statistically significant. (TIF) [file pone.0248111.s002.tif]

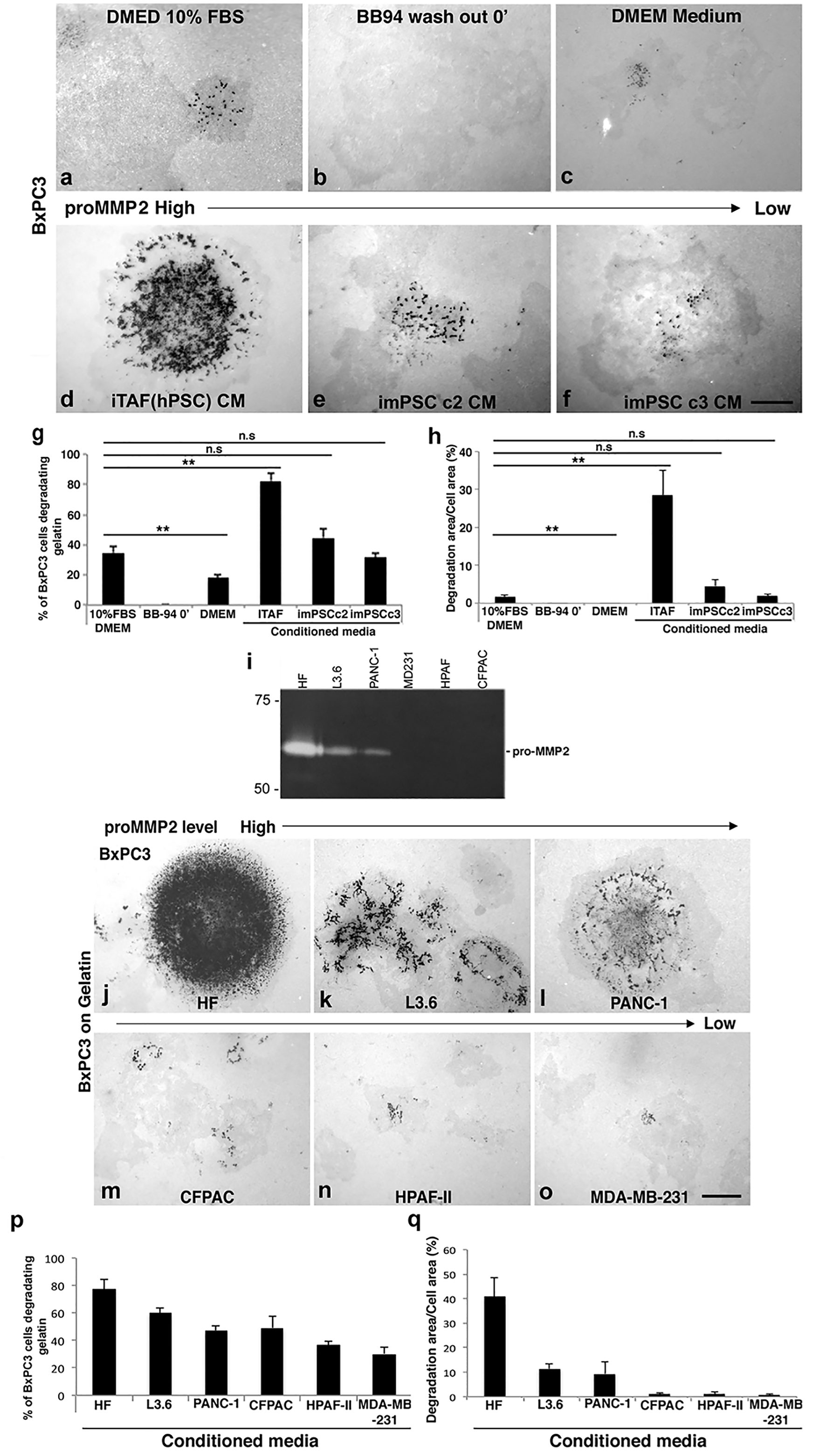

Supplement: S3 Fig — (a-h) Fluorescence images of BxPC3 tumor cells seeded on green fluorescent gelatin-coated coverslips and cultured with (a) DMEM containing 10% FBS for 8 h before fixation, (b) the MMP inhibitor BB-94 (2 μM) for 8 h, (c-f) CM from different stromal cells for 8 h after BB-94 washout. (g,h) Graphs depicting matrix degradation by cells described above and quantified as either the percentage of BxPC3 cells degrading matrix (≥100 cells per condition, g) or the degradation area per cell area (≥10 cells per condition, h). These data suggest a direct correlation between the level of MMP2 in the stromal cell CM and the matrix degradation induced by the addition of this CM to the BxPC3 cells. Scale bar = 10μm. Bar graph represents averages ± SEM from 3 independent experiments. **p <0.01. n.s, not statistically significant. (i-q) MMP2 levels in CM collected from cancer cells correlates with the capacity to promote matrix degradation in recipient BxPC3 cells. (i) Zymography demonstrating MMP2 activity in 5 different PDAC cell lines compared to HFs. (j-o) Representative images of BxPC3 degrading matrix in response to CM derived from other tumor cells represented in the above zymogram. Note that the tumor cells secreting the lower amounts of MMP2 into the CM induce the least degradation by the recipient BxPC3 cells. Scale bar = 10μm. (p,q) Bar graph quantifying BxPC3 matrix degradation in response to CM from the distinct tumor cells. The percentage of BxPC3 cells degrading matrix was determined with ≥100 cells per condition and the degradation area per cell area was quantified with ≥10 cells per condition. Graphs represent averages ± SEM from 3 independent experiments. (TIF) [file pone.0248111.s003.tif]

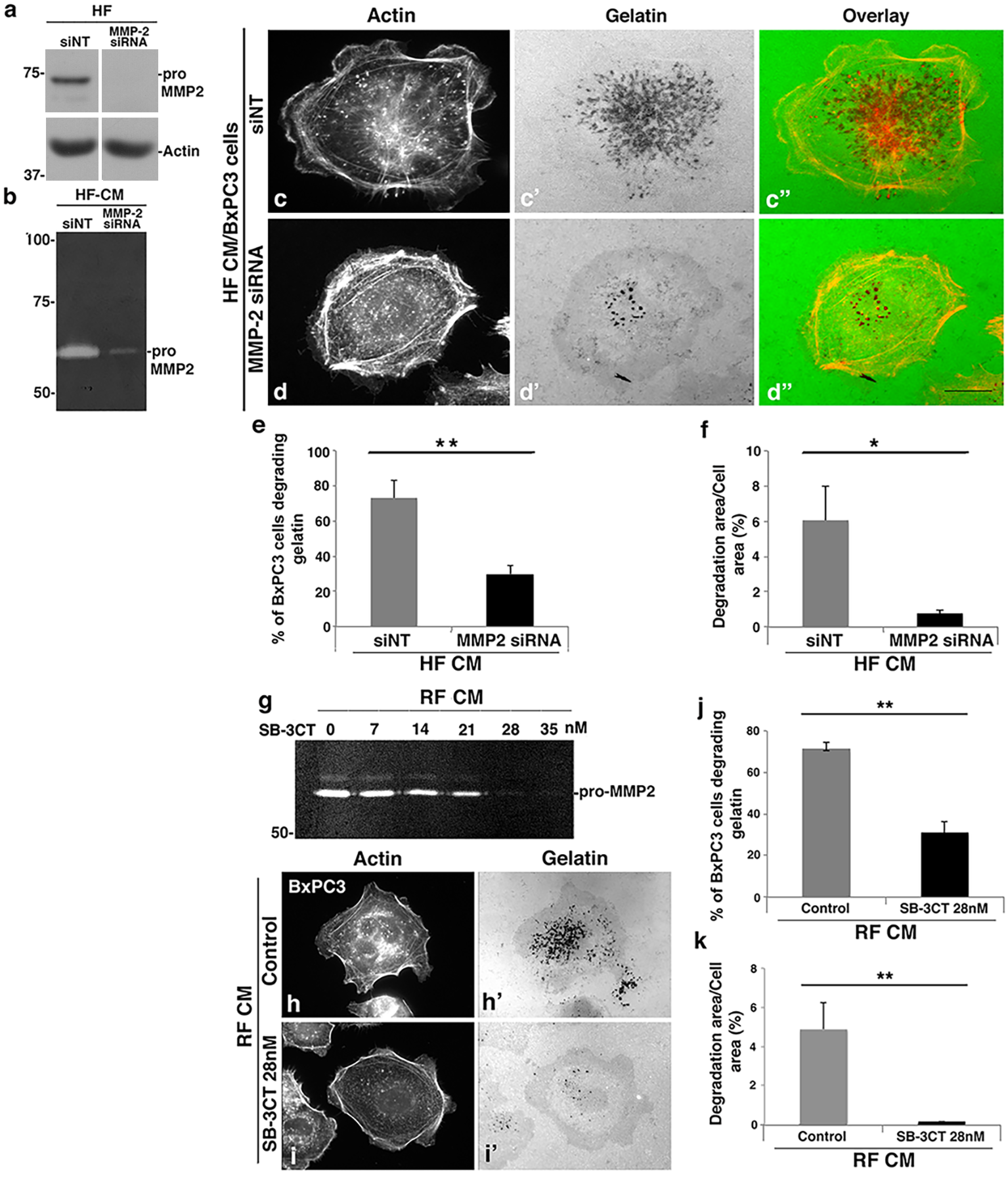

Supplement: S4 Fig — (a) Western blot of HF cells treated with control siRNAs or siRNAs to reduce MMP2 levels. (b) Zymogram showing loss of MMP2 in the siRNA-treated cells described in (a). (c-d”) Fluorescence images of BxPC3 cells plated on green fluorescent gelatin-coated coverslips and incubated 8 h with CM collected from HFs cells treated with control siRNAs or siRNAs to reduce MMP2 levels. Phalloidin staining of actin was used to show cell borders (c, d). Scale bar = 10μm. (e,f) Quantitation of the experiment described above. Depletion of MMP2 from the HF-CM reduced the number of BxPC3 cells degrading matrix (≥100 cells per condition) and the total area of degraded substrate per cell compared to controls (≥10 cells per condition). (g) Zymogram showing a dose curve of the MMP2 inhibitor SB-3CT, when added to RFs, on MMP2 for 24 h prior to CM collection. (h,h’-i, i’) Fluorescence images of BxPC3 cells seeded on green fluorescent gelatin-coated coverslips and cultured in RF-CM with or without 28 nM of the SB-3CT inhibitor added for 8 h. (j,k) The MMP2 inhibitor reduced the percent of cells degrading matrix by 3 fold (≥100 cells per condition, j), and the area degraded per cell in BxPC3 cells by 30 fold (≥10 cells per condition, k). Graphs represent averages ± SEM from at least 3 independent experiments. *p<0.05, **p <0.01. (TIF) [file pone.0248111.s004.tif]

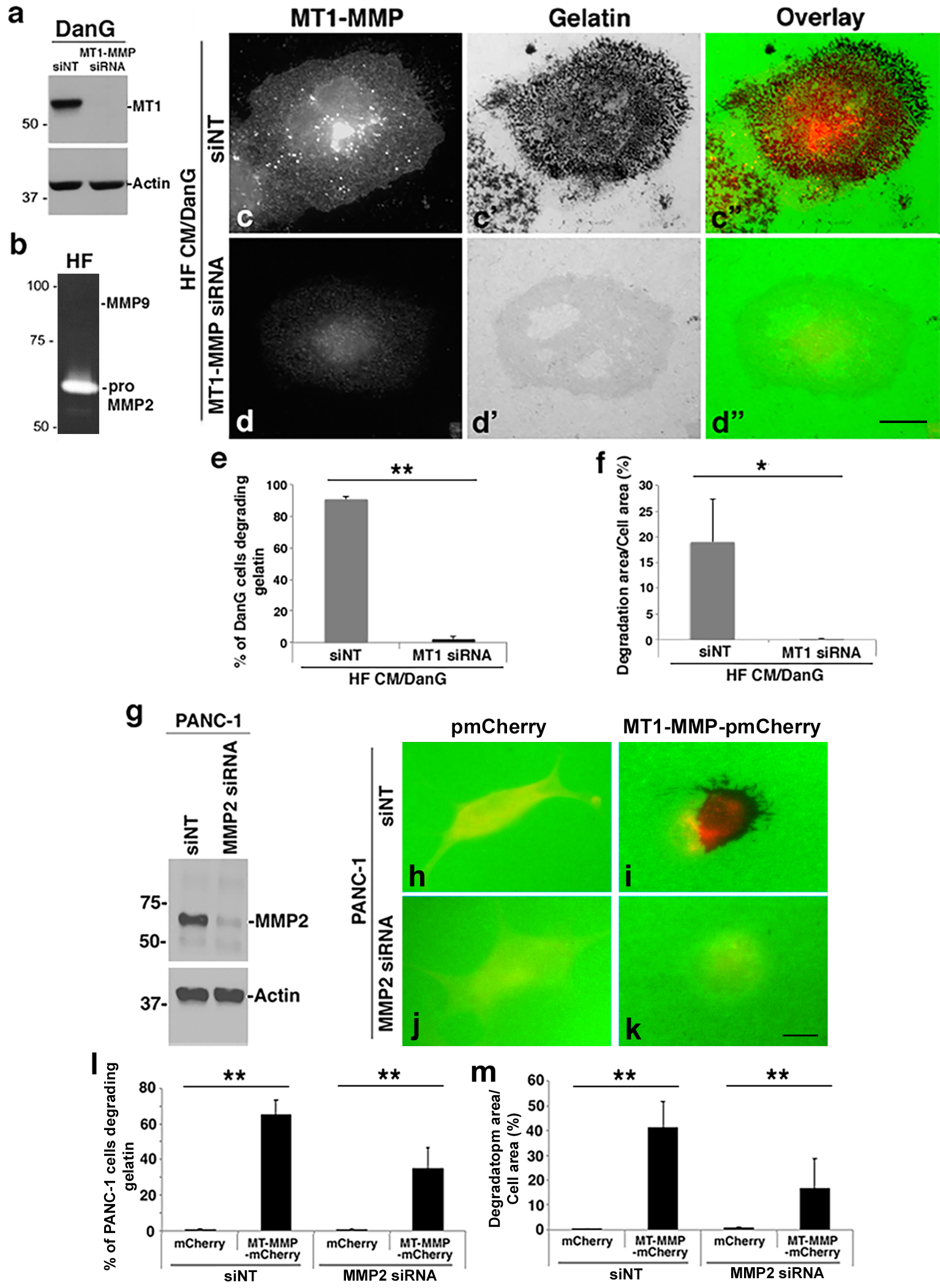

Supplement: S5 Fig — MT1-MMP knockdown in DanG cells inhibits matrix degradation in the presence of HF CM. (a) DanG cells were transfected with control siRNAs or siRNA targeting MT1-MMP. Knockdowns were confirmed by western blotting. (b) The presence of MMP2 in HF CM was tested by zymography. (c-d”) Fluorescence micrographs of DanG cells that were transfected with control or siRNAs against MT1-MMP for 3 days and then seeded onto green fluorescent gelatin-coated coverslips and cultured in CM from HF cells for 8 h. The level of MT1-MMP in individual cells was tested by immunofluorescence using antibodies against MT1-MMP. Scale bar = 10μm. (e-f) Matrix degradation was quantified, showing that MT1-MMP depletion led to a substantial decrease both in the percent of cells degrading matrix (≥100 cells per condition, e) and in the area degraded per cell in DanG cells (≥10 cells per condition, f). (g) PANC-1 cells were transfected with control siRNAs or siRNA targeting MMP2. Knockdown was confirmed by western blotting. (h-k) Control or MMP2-depleted PANC-1 cells were transfected with mCherry vector (h,j) or mCherry MT1-MMP (i,k) and plated on fluorescent gelatin-coated coverslips. Matrix degradation was quantified, showing that MT1-MMP overexpression led to a substantial increase both in the percent of cells degrading matrix (>100 cells per condition, l) and the area of matrix degradation (> 10 cells per condition, m), which was partially suppressed by knockdown of MMP2. Graphs represent averages ± SEM from at least 3 independent experiments. *p <0.05. **p <0.01. (TIF) [file pone.0248111.s005.tif]

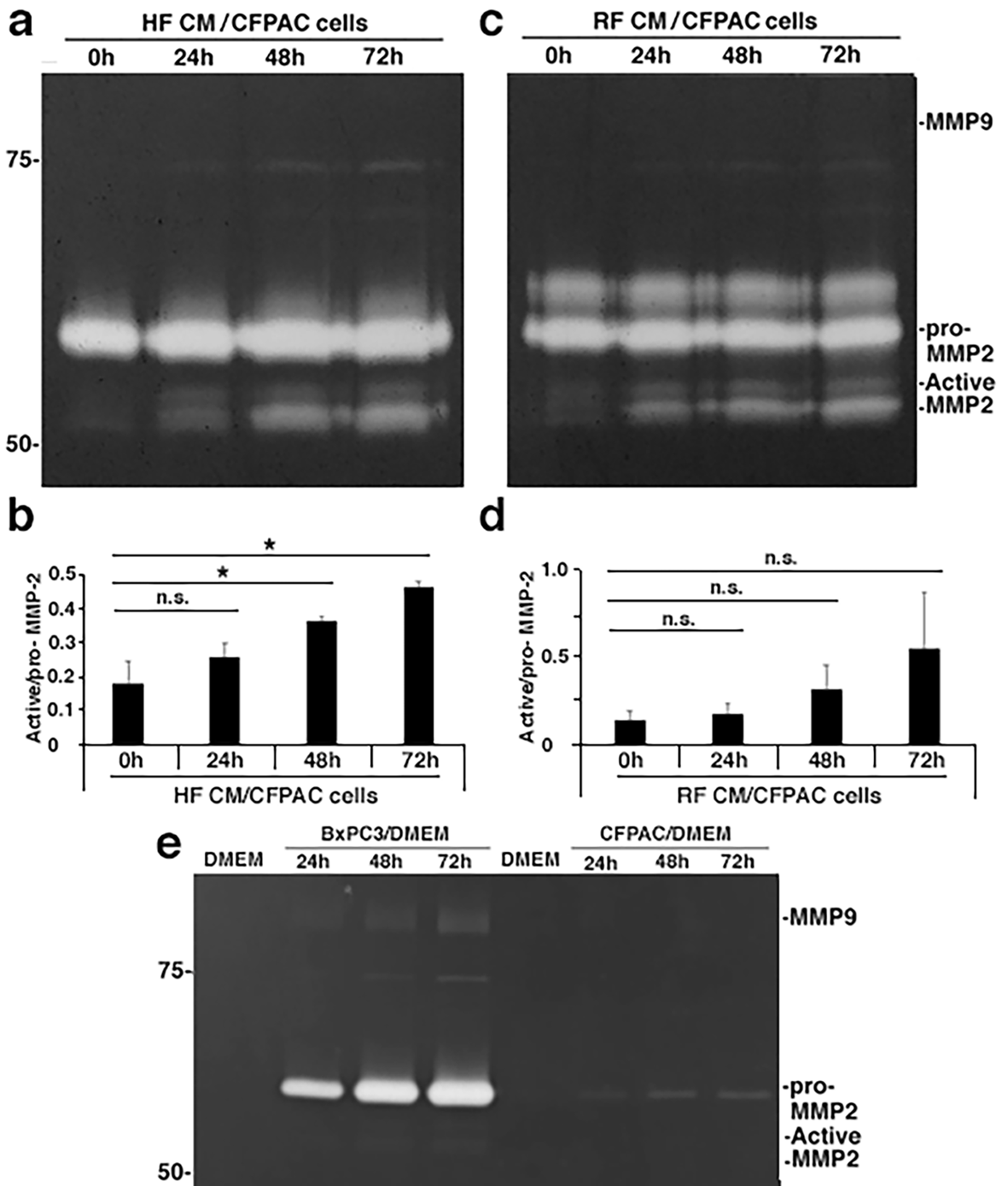

Supplement: S6 Fig — (a-d) CM from HFs or RFs was collected and applied to CFPAC cells. The CM from CFPAC cells were then further collected after 0, 24, 48 or 72 h and subjected to zymography. (a, c) Representative zymograms showing MMP2 activity of the CM described above. The amount of active MMP2 from the HF-CM (a) or RF-CM (c) was increased with longer incubation times with CFPAC cells. (b, d) Quantitation of the ratio of active to pro-MMP2 from the zymograms of 3 independent experiments. Data represent averages ± SEM. *p <0.05. **p <0.01. n.s, not statistically significant. (e) CM from BxPC3 cells and CFPAC cells were collected at 24, 48 or 72 h and subjected to zymography. (TIF) [file pone.0248111.s006.tif]
