# Supplementary material for: Synergistic metalloproteinase-based remodeling of matrix by pancreatic tumor and stromal cells
Source: PLoS One. 2021 Mar 19;16(3):e0248111. doi: 10.1371/journal.pone.0248111 (PMC7978280; doi:10.1371/journal.pone.0248111)
Supplement: S1 Raw images — (PDF) [file pone.0248111.s007.pdf]

Fig.3a

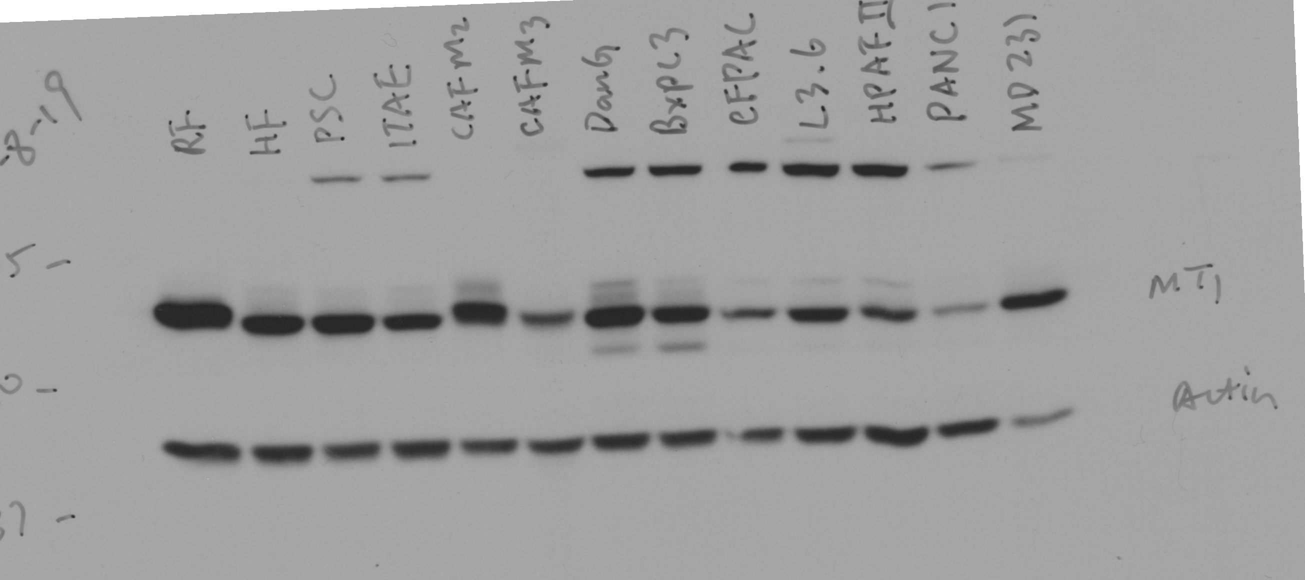

CanoScan LiDE120

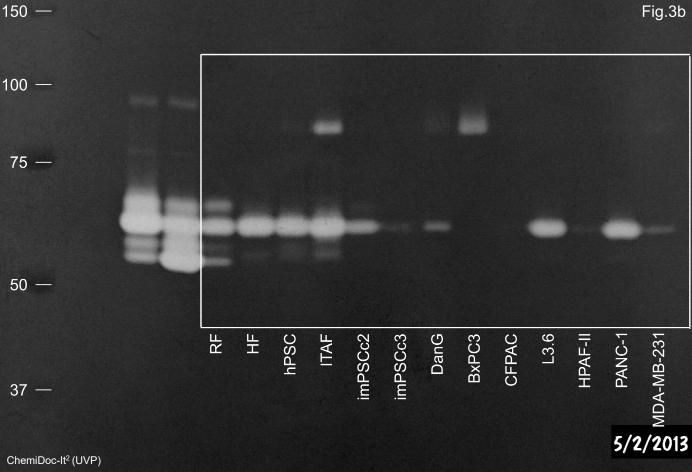

Fig.3b
150
100
75
50
37
RF
HF
hPSC
ITAF
imPSCc2
imPSCc3
DanG
BxPC3
CFPAC
L3.6
HPAF-II
PANC-1
MDA-MB-231
5/2/2013
ChemiDoc-It² (UVP)

Fig.3c

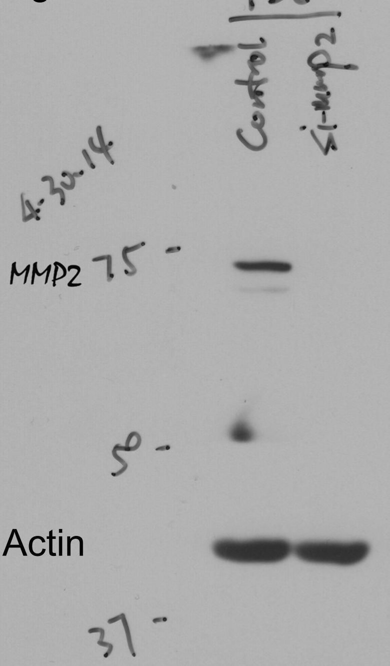

CanoScan LiDE120

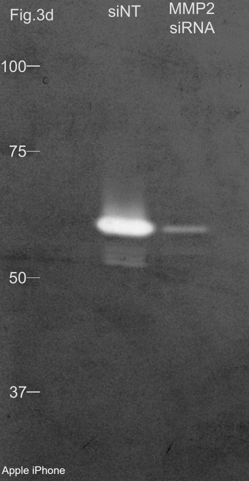
Fig.3d
siNT
MMP2
siRNA
100
75
50
37
Apple iPhone

Fig.4a

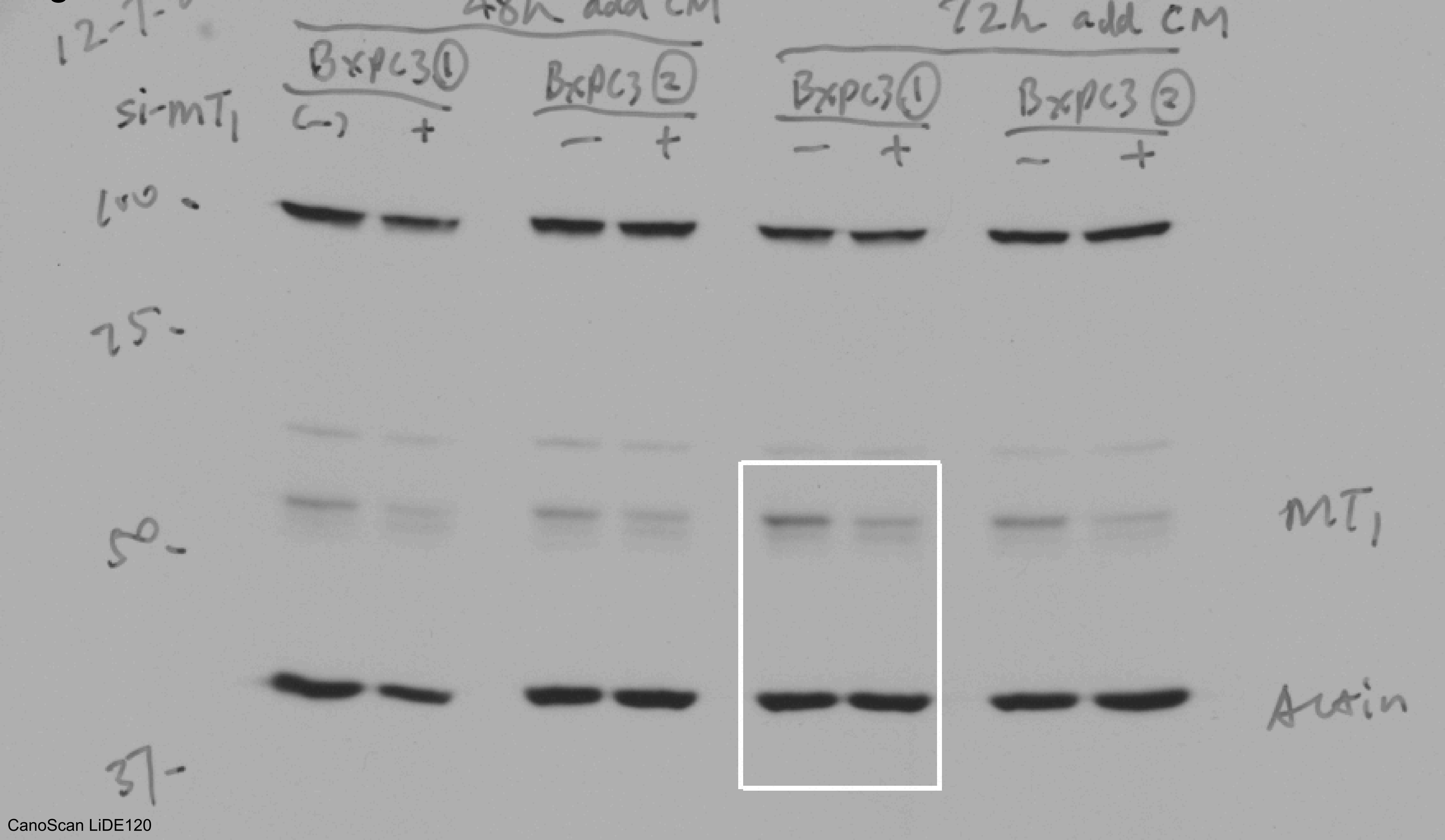
12-7-20
48h add CM
72h add CM
Bxpc3 1
Bxpc3 2
si-MT1
(-) +
- +
100
75
50
37
MT1
Actin
CanoScan LiDE120

Fig.4c (left)

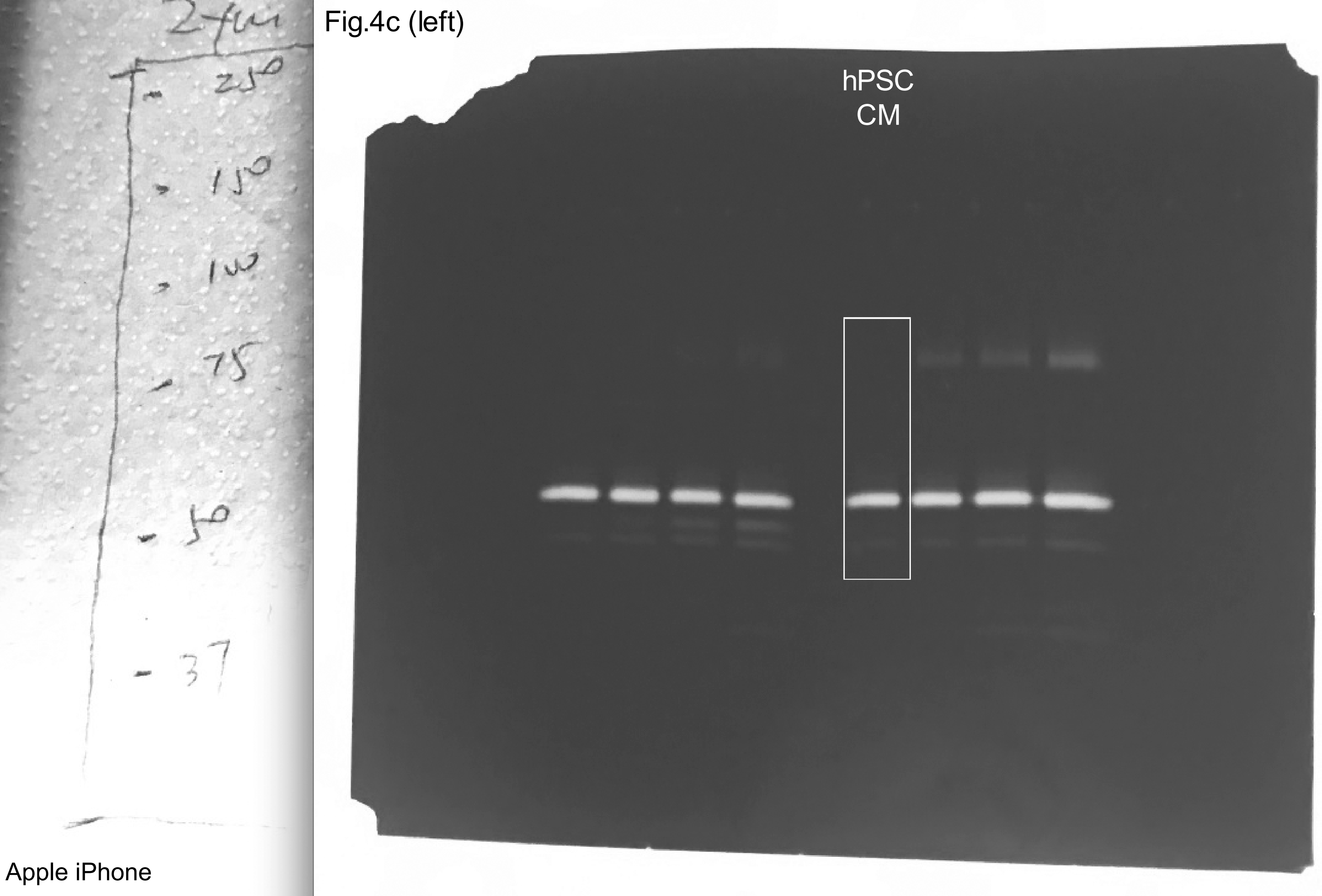

Apple iPhone

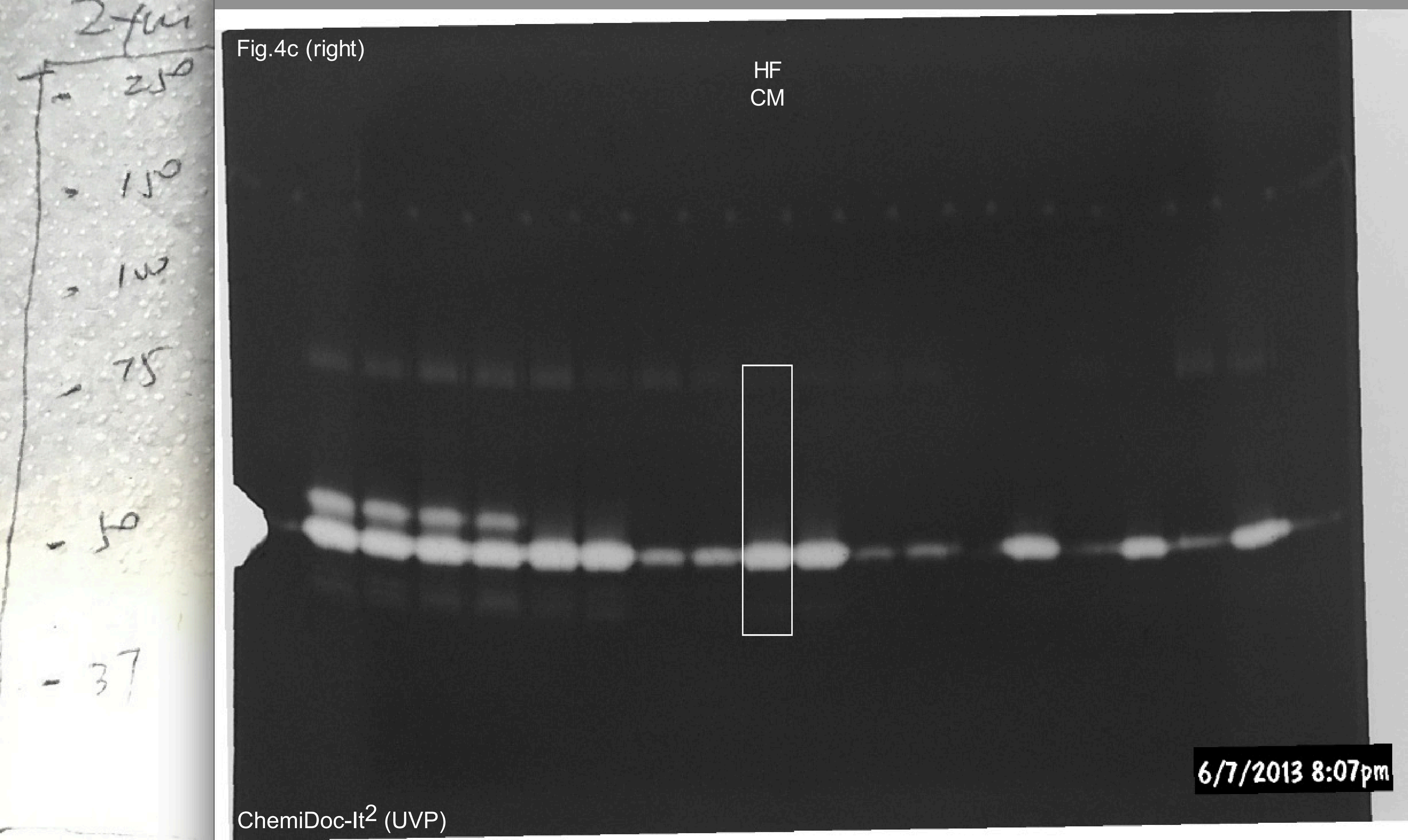
Fig.4c (right)
HF
CM
250
150
100
75
50
37
6/7/2013 8:07pm
ChemiDoc-It2 (UVP)

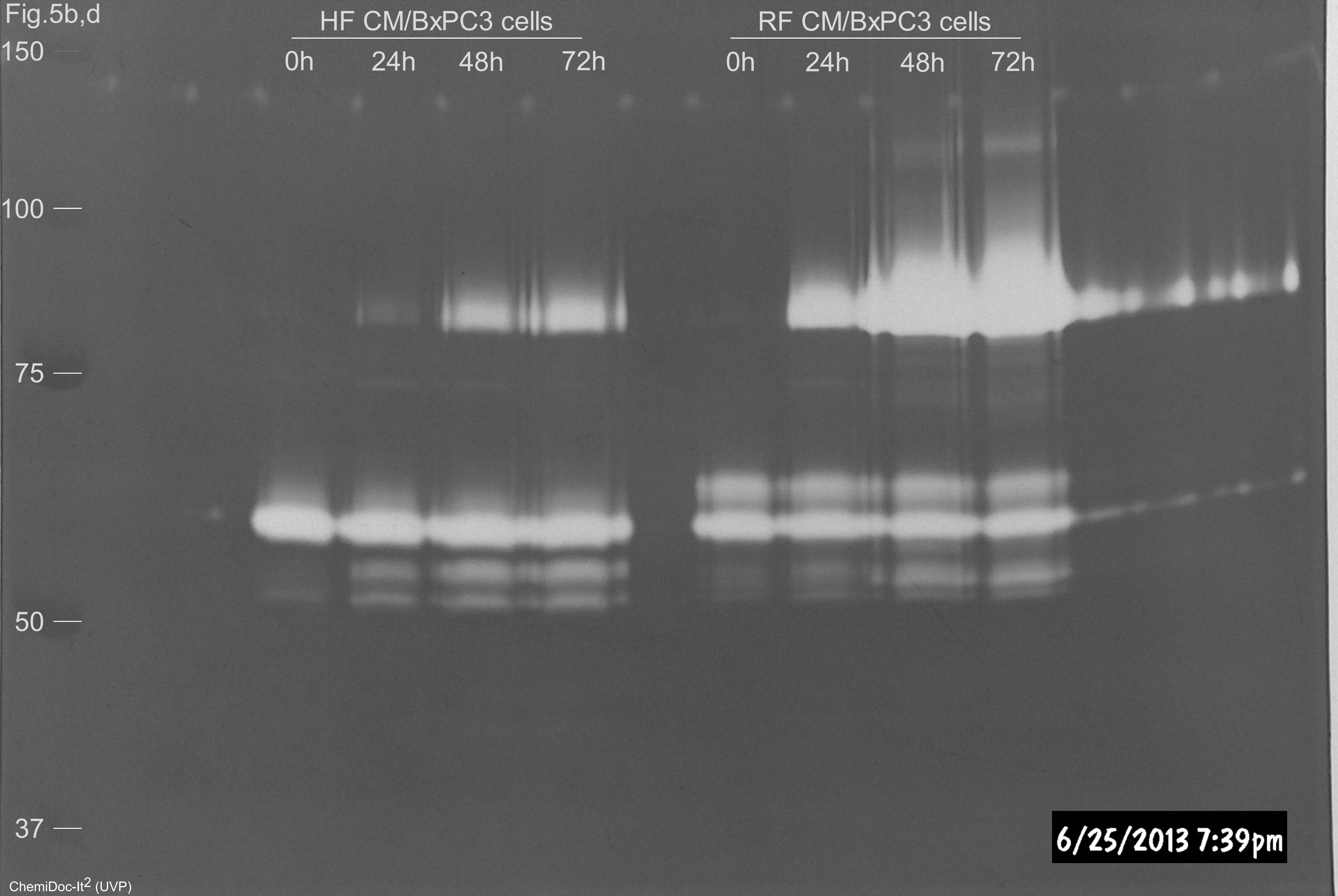
Fig.5b,d
HF CM/BxPC3 cells
RF CM/BxPC3 cells
0h
24h
48h
72h
0h
24h
48h
72h
150
100
75
50
37
6/25/2013 7:39pm
ChemiDoc-It2 (UVP)

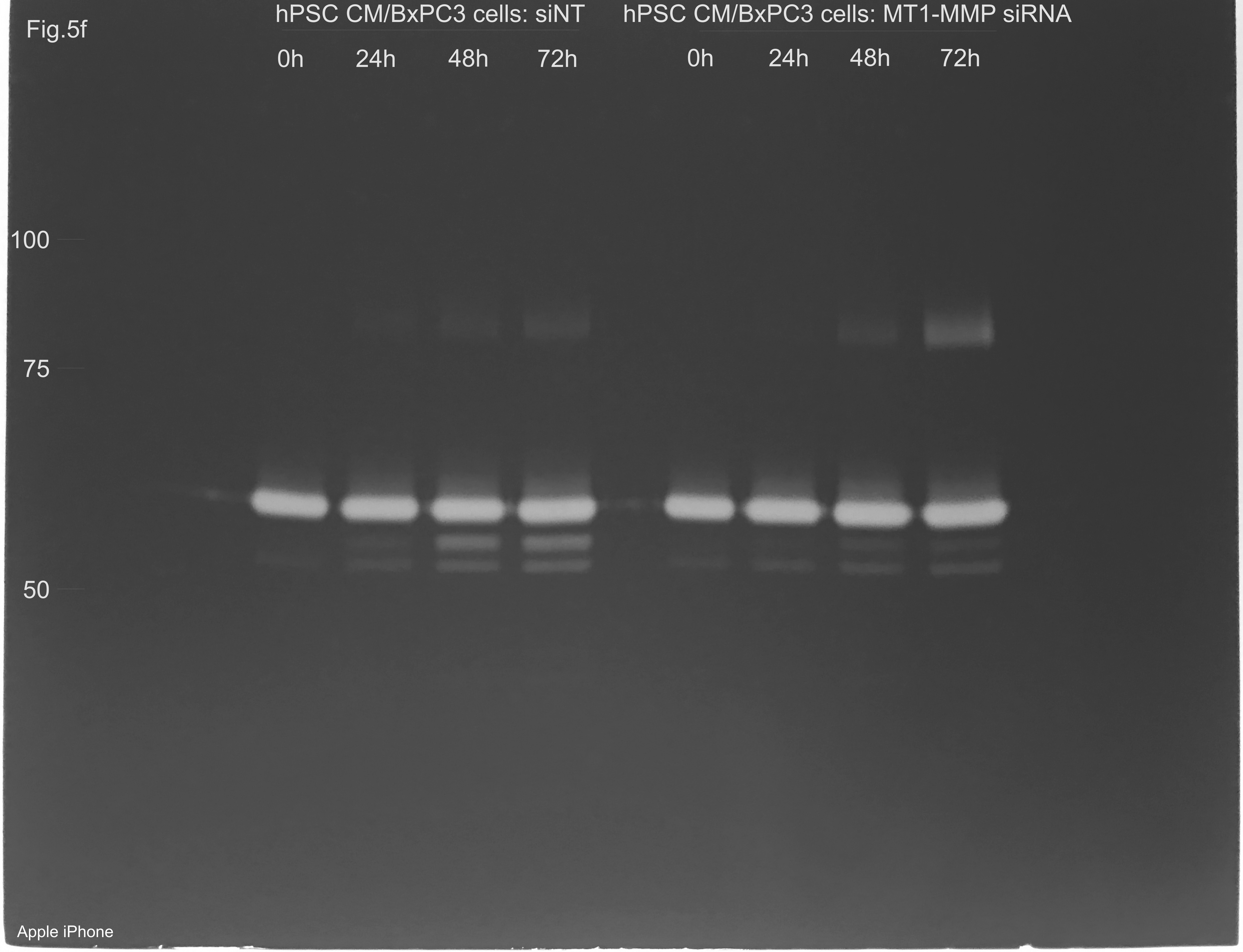
Fig.5f
hPSC CM/BxPC3 cells: siNT
hPSC CM/BxPC3 cells: MT1-MMP siRNA
0h
24h
48h
72h
0h
24h
48h
72h
100
75
50
Apple iPhone

Fig.S3i

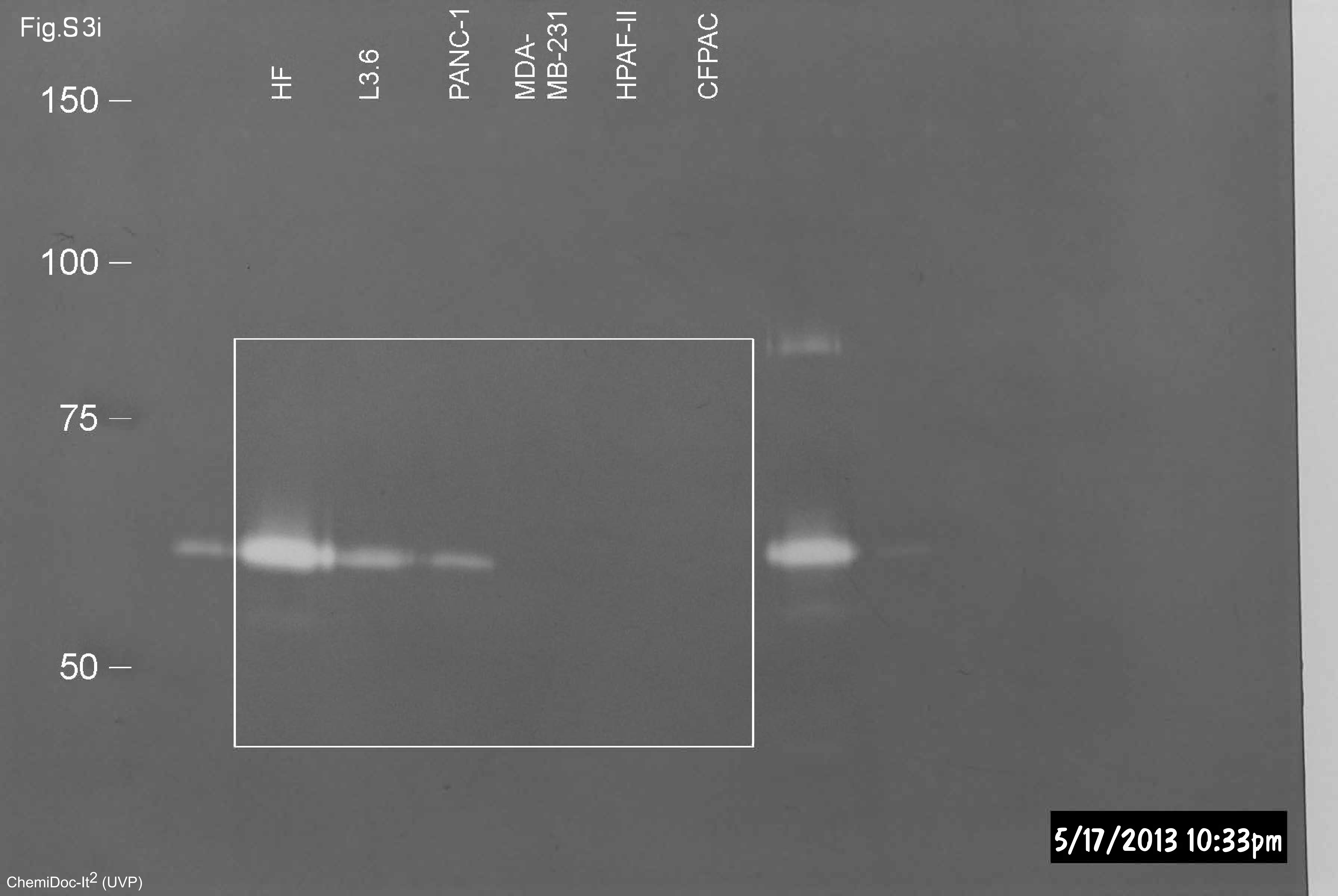

150
100
75
50
HF
L3.6
PANC-1
MDA-MB-231
HPAF-II
CFPAC
5/17/2013 10:33pm
ChemiDoc-It2 (UVP)

Fig.S4a

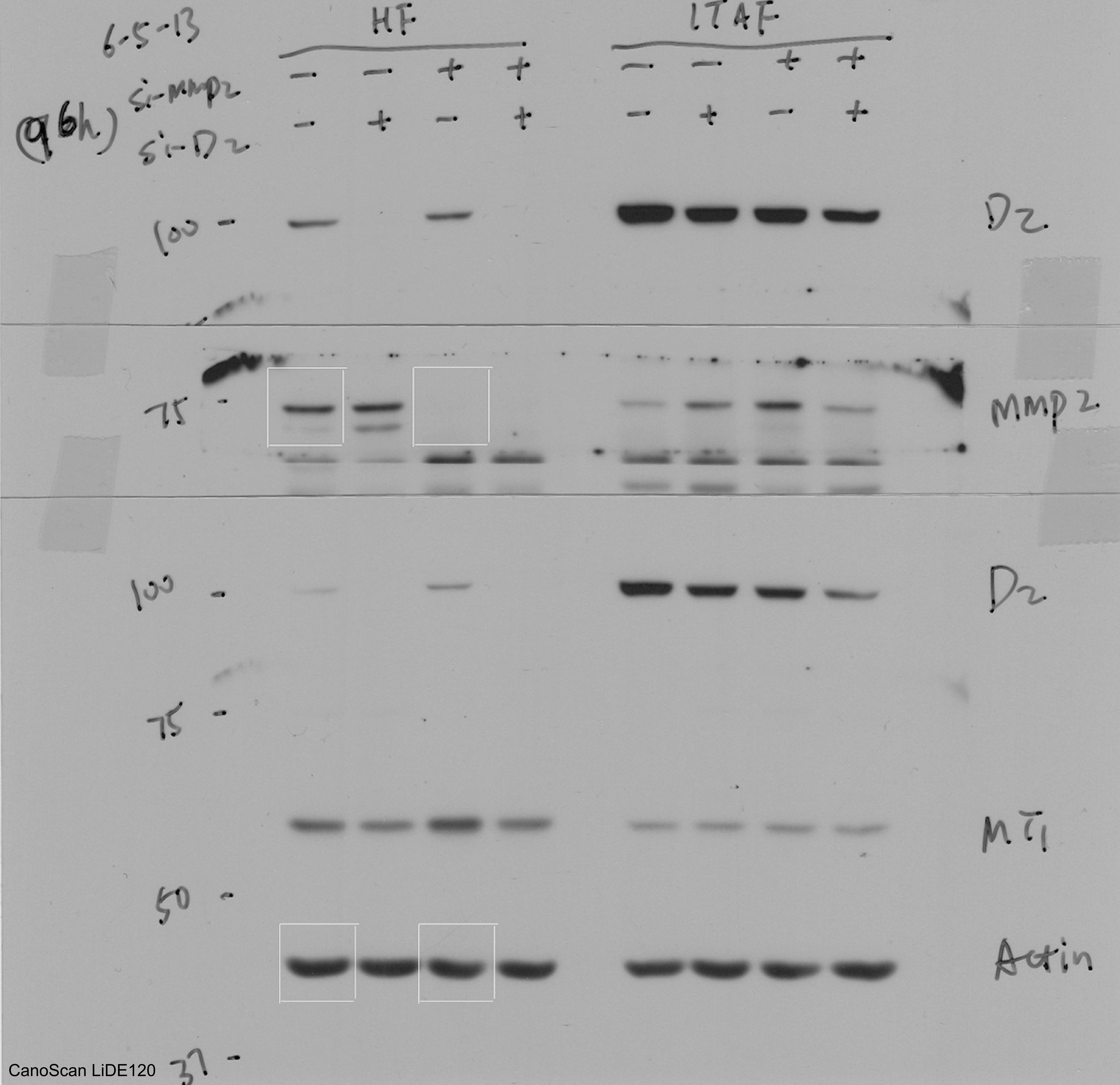
6-5-13
HF
LTAF
si-MMP2
si-D2
(96h)
100
75
50
37
D2
MMP2
MT1
Actin
CanoScan LiDE120

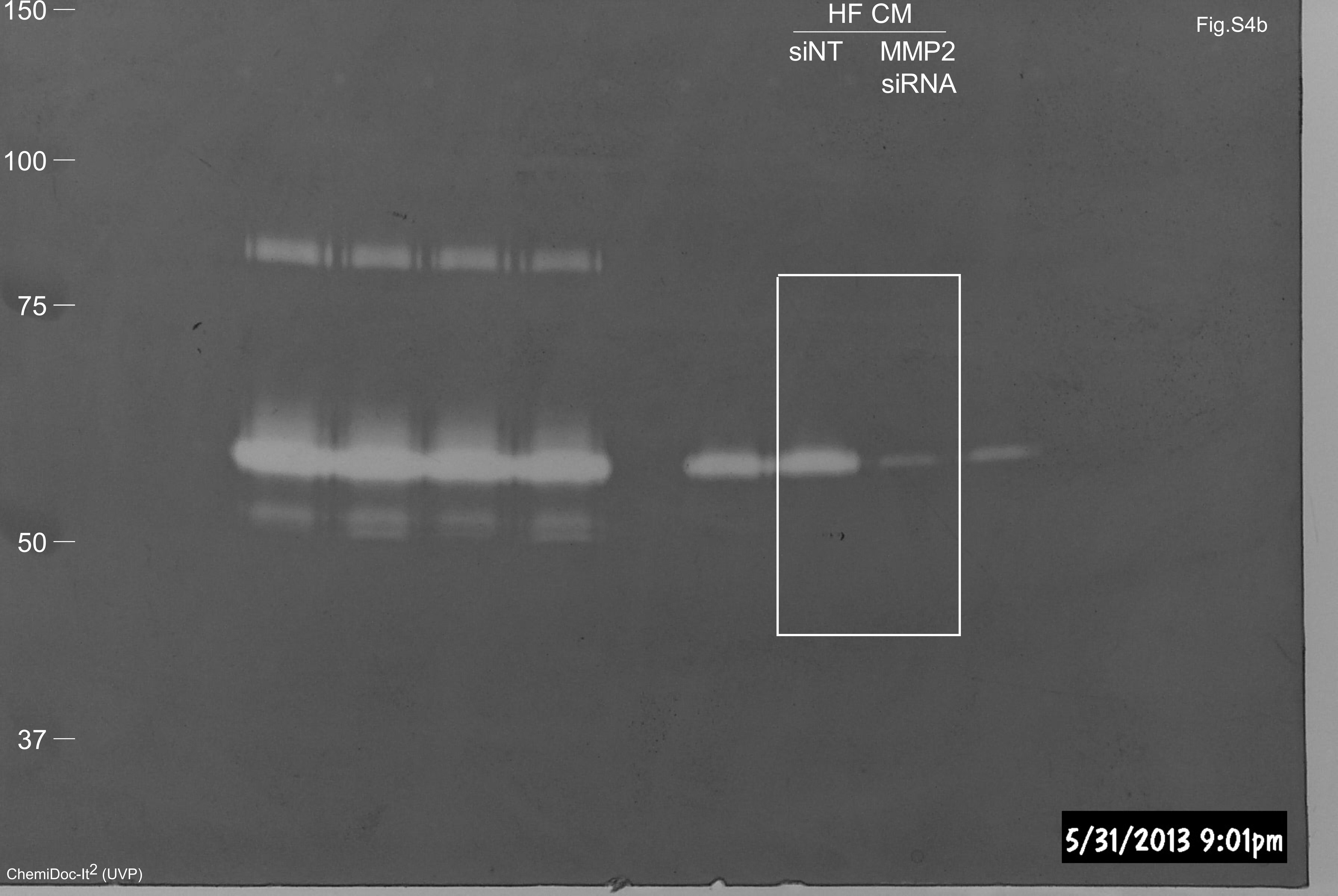
Fig.S4b
HF CM
siNT
MMP2 siRNA
150
100
75
50
37
5/31/2013 9:01pm
ChemiDoc-It2 (UVP)

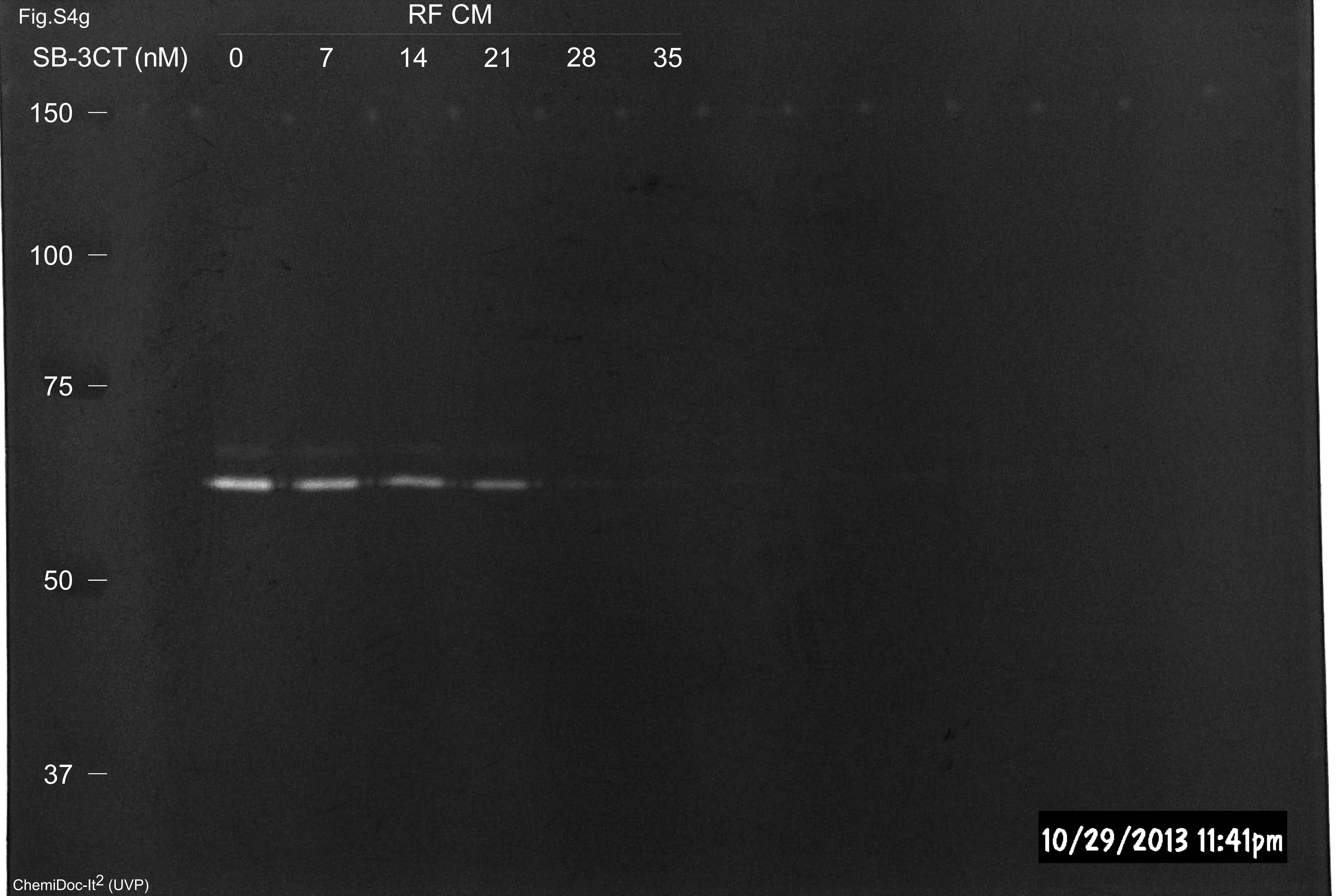
Fig.S4g
RF CM
SB-3CT (nM)
0
7
14
21
28
35
150
100
75
50
37
10/29/2013 11:41pm
ChemiDoc-It2 (UVP)

Fig.S5a

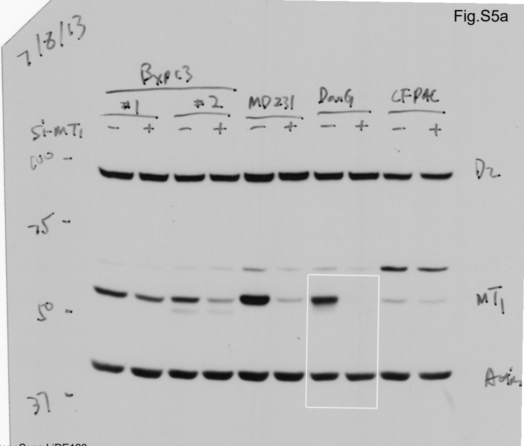

CanoScan LiDE120

Fig.S5b

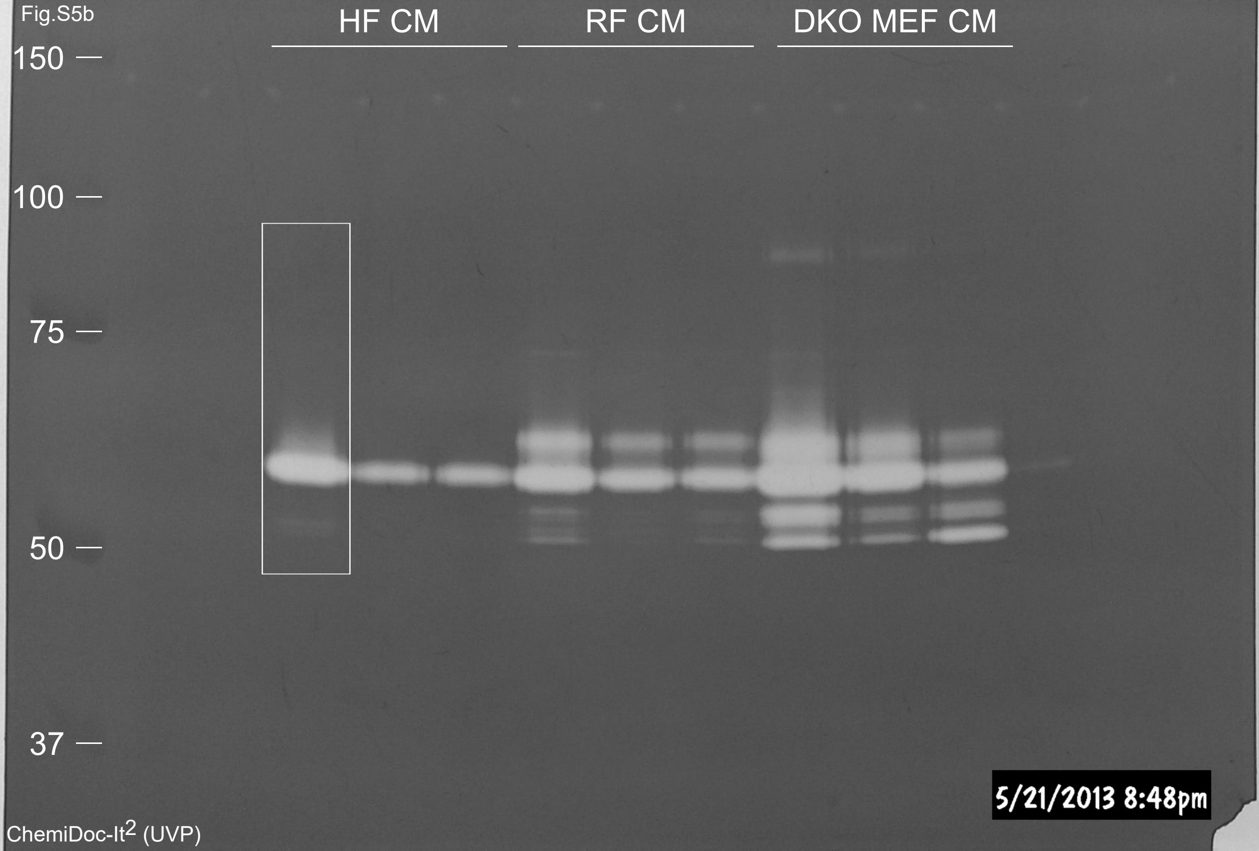
HF CM
RF CM
DKO MEF CM
150
100
75
50
37
5/21/2013 8:48pm
ChemiDoc-It2 (UVP)

Fig.S5g

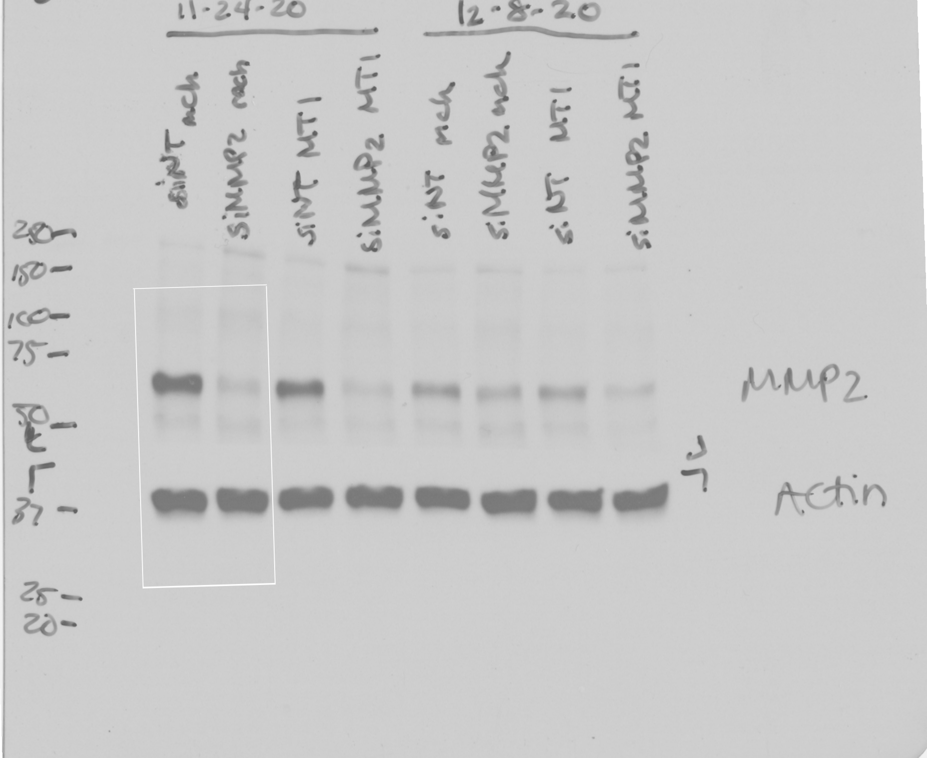

CanoScan LiDE120

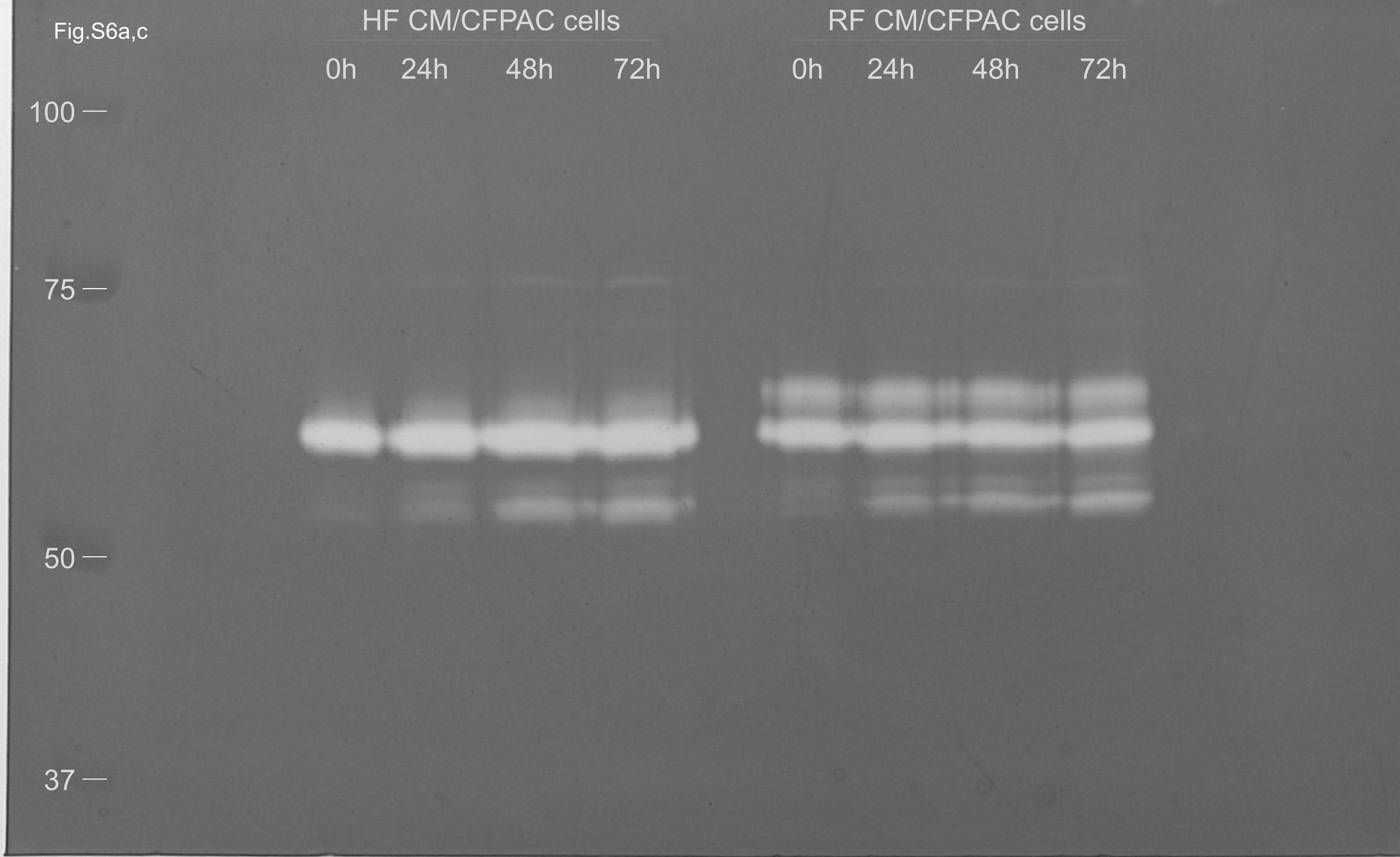

ChemiDoc-It^2 (UVP)

6/26/2013 8:30pm

Fig.S6e

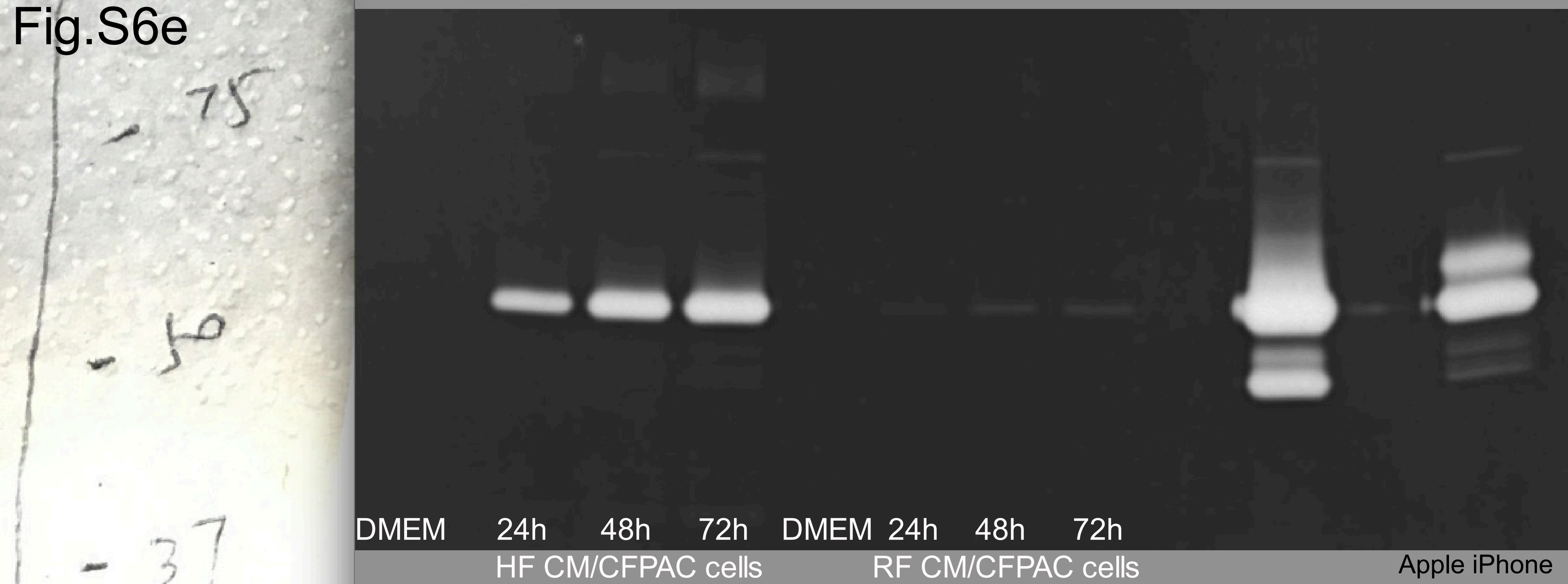
75
50
37
DMEM
24h
48h
72h
DMEM
24h
48h
72h
HF CM/CFPAC cells
RF CM/CFPAC cells
Apple iPhone
